# Cost-effectiveness of osteoporotic fracture risk assessment in people with intellectual disabilities: a UK NHS modelling study

**DOI:** 10.1136/bmjopen-2025-110008

**Published:** 2026-04-17

**Authors:** May Ee Png, Valeria Frighi, Tim Adrian Holt, Felix Achana, Margaret C Smith, Gary Stephen Collins, Jan Roast, Stavros Petrou

**Affiliations:** 1 Nuffield Department of Primary Care Health Sciences, University of Oxford, Oxford, UK; 2 Department of Psychiatry, University of Oxford, Oxford, England, UK; 3 Oxford Health NHS Foundation Trust, Oxford, UK; 4 Department of Applied Health Sciences, School of Health Sciences, College of Medicine and Health, University of Birmingham, Birmingham, England, UK; 5 NIHR Birmingham Biomedical Research Centre, University Hospitals Birmingham NHS Foundation Trust, Birmingham, England, UK; 6 Dimensions UK, Reading, UK

**Keywords:** HEALTH ECONOMICS, Fractures, Bone, Preventive Health Services, General Practice, Vulnerable Populations

## Abstract

**Abstract:**

**Objectives:**

We compared the cost-effectiveness of alternative fracture risk assessment strategies for people with intellectual disabilities (ID) aged ≥40 years from a UK National Health Services perspective over a lifetime horizon.

**Design:**

Cost-effectiveness analysis using a lifetime decision-analytical model.

**Setting:**

UK primary care, with data from literature and national databases.

**Participants:**

People with ID.

**Interventions:**

Three strategies were assessed: (S1) Risk assessment using the UK QFracture score; (S2) use of IDFracture (a fracture risk prediction tool specifically developed for adults with ID); and (S3) conducting a one-time dual-energy X-ray absorptiometry (DXA) scan in all. S1 and S2 were followed by DXA scan for those at risk. At-risk individuals received treatment according to UK practice (bisphosphonates plus vitamin D and calcium for osteoporosis, and vitamin D and calcium alone for osteopenia).

**Primary outcome measures:**

Direct healthcare costs and quality-adjusted life years (QALYs), and incremental cost-effectiveness ratio (ICER).

**Results:**

In the base case, S2 (ICER: −£2568/QALY) was dominant (ie, less costly and more effective) and S3 (ICER: £1678/QALY) was cost-effective relative to S1 for major osteoporotic fracture (MOF). For hip fracture, S2 (ICER: £32 116/QALY) and S3 (ICER: £49 536/QALY) were not cost-effective relative to S1 under the National Institute for Health and Care Excellence-recommended cost-effectiveness thresholds. Findings from the sensitivity analyses were predominantly consistent with the base-case results. Subgroup analyses showed that age-specific and gender-specific strategies could be used.

**Conclusion:**

For people with ID aged ≥40 years, a proactive approach to risk assessment for MOF is not only clinically beneficial, but also cost-effective.

**STRENGTHS AND LIMITATIONS OF THIS STUDY**

- We assessed a large UK health database drawing on real-world data from both primary and secondary care to conduct this health economic analysis of fracture risk assessment.
- Osteoporosis and osteopenia were modelled as distinct health states, allowing differential treatment pathways and transition probabilities.
- Several model parameters were derived from non-intellectual disability populations due to limited availability of intellectual disability-specific data.
- Key assumptions, including a healthcare system perspective, single baseline risk assessment, lifetime treatment, full dual-energy X-ray absorptiometry (DXA) uptake, linear extrapolation of fracture risk and use of an earlier QFracture version may limit generalisability.
- The benefits of fracture risk assessment may have been underestimated as only those with osteoporosis on DXA were assumed to be treated with antiresorptive therapy.

##  Introduction

Based on a 2006 estimate of the global burden of osteoporotic fracture,^1^ there were around 9 million osteoporotic fractures annually (or 1 occurring every 3 s^2^). Osteoporotic fractures are associated with a reduction in quality of life, loss of functional independence, higher risk of mortality and higher economic costs.^3^ This burden has undoubtedly grown over the years due to an ageing population and although not estimated, would be particularly significant among people with intellectual disabilities (ID), as the incidence of major osteoporotic fractures (MOF) and hip fractures (HF) is higher than the general population.^4^

Osteoporosis, a silent disease that may remain asymptomatic until fracture occurs, can be detected via a dual-energy X-ray absorptiometry (DXA), also known as the bone mineral density (BMD) scan, and treated with bone strengthening medicines. Current practice in the UK based on National Institute for Health and Care Excellence (NICE) and National Osteoporosis Guideline Group guidelines^5^ recommends use of a risk score known as ‘QFracture’ to be used to estimate the 10-year absolute risk of MOF and HF in the primary care setting. However, people with ID do not generally have their fracture risk assessed, and when they do, the standard risk scores are poorly tailored to this population. Their risk, therefore, goes unrecognised, and few are offered the opportunity to prevent fractures. A new fracture risk prediction tool (IDFracture) for assessing fracture risk in people with ID has been developed and validated (Smith *et al*, in press).^6^ IDFracture is an algorithm that generates a risk score estimating the 10-year risk of MOF and of HF for adults aged 30–79 years. Although recommended by NICE,^5^ FRAX (Fracture risk assessment tool) was not considered in this study because QFracture was favoured over FRAX in its more recent documentation on osteoporosis.^7^

We aimed to compare the cost-effectiveness of three potential fracture risk assessment strategies for MOF and HF among people with ID aged 40 years and above from the UK National Health Service (NHS) perspective over a lifetime horizon.

## Methods

The results are reported in accordance with the Consolidated Health Economic Evaluation Reporting Standards 2022 statement^8^ for health economic evaluations.

### Fracture risk assessment strategies

The three fracture risk assessment strategies examined were:

1. Current practice based on guidance from NICE, entailing screening via QFracture^9^ for those deemed at risk by age and/or other criteria, followed by DXA for those above a treatment intervention threshold;^7 10^
2. Fracture risk assessment via IDFracture for all aged 40 years and above, followed by DXA for those above a treatment intervention threshold; and
3. DXA alone for all aged 40 years and above.

QFracture was selected to determine eligibility for DXA in Strategy 1 to reflect its use within UK primary care systems and its inclusion in NICE fracture risk assessment guidance at the time of study design.^11^ Although FRAX is also widely used, the objective of this analysis was to evaluate strategies aligned with UK NHS practice.

### Model structure

A decision tree with a Markov cohort model was constructed to reflect the options of the three fracture risk assessment strategies, in the primary care setting while modelling recurring outcomes through time to assess its costs and effects among those aged 40 years old from a UK NHS perspective over a lifetime horizon. The age of 40 years was chosen because our previous study^12^ indicated that this is the age at which people with ID would start to develop significant risk of osteoporotic fracture.

In the decision tree (online supplemental figure 1), based on the current NICE criteria for determining risk of MOF and HF using QFracture, we estimated that approximately 3% (1%–5%) of the population with ID would be screened at age 40–49 years. Those who tested positive (true and false positives) using the QFracture or IDFracture risk scores and were therefore considered at risk of fracture were treated according to their BMD T-scores after a diagnostic DXA. The treatment intervention threshold of testing positive for MOF was defined as at least 10% over 10 years, and at least 3% over 10 years for HF.^5^ NICE does not offer specific guidance but advises clinicians to follow local protocols or other national guidelines for advice on intervention thresholds. Therefore, our choice was based on the most recent recommendations from the Scottish Intercollegiate Guidelines Network, updated in 2021 for MOF.^13^ For HF, our choice was based on the 2021 clinical guideline for the prevention and treatment of osteoporosis by the National Osteoporosis Guideline Group,^5^ which sets the intervention threshold at 2.3% and 3.5% 10-year risk at age 60 and 65 years, respectively.

The study cohort consisted of people aged 40–79 with ID, who were registered at their current practice at some point between 1 January 2008 and 31 October 2020 and were eligible for linkage to the Hospital Episode Statistics (HES)^14^ data and Index of Multiple Deprivation. The modelled cohort had a mean age of 51.9 years and comprised 50% men and 50% women. The HES dataset contains hospital records for the majority of patients attending secondary care services within the NHS in England. The diagnostic and service user codes used to identify people with ID have been described elsewhere.^4^ Individuals above 79 years old were excluded because of the small number of individuals in this age group. We estimated the 10-year risk of fracture by applying the IDFracture prediction models^6^ to a cohort of people with ID, extracted from the Clinical Practice Research Datalink (CPRD) Aurum database. The CPRD Aurum captures data from over 40 million patients with 13 million current patients (20% of the UK population) over 1000 general practices using EMIS Web software.^15 16^ We also applied a modified version of the QFracture 2012 algorithms^9^ to these data.

Based on the DXA, people who had a T-score of 2.5 SD or more below the young adult mean were considered as having osteoporosis, those with a T-score that was between 1–2.5 SD below the young adult mean were considered as having osteopenia, while those with BMD within 1 SD (+1 or −1) of the young adult mean were considered as having normal BMD.^17^ Those with osteoporosis were treated using bisphosphonates in addition to vitamin D and calcium, while those with osteopenia were treated with vitamin D and calcium alone in line with common UK practice.^18^ This approach excluded the additional step of re-estimating fracture risk using BMD values obtained from the DXA, which would have required too many assumptions about these values in the ID population. Since the 2017 NICE guidance noted that using the lowest available cost for each bisphosphonate in the cost-effectiveness modelling reflected clinical practice and would be appropriate,^19^ alendronate 10 mg daily was selected among the bisphosphonates as the treatment of choice. We selected colecalciferol at a dose of 800 units and calcium at a dose of 1000 mg in view of the high prevalence of vitamin D deficiency in people with ID^20^ and of the standard calcium supplementation dose. All treatments were assumed to be given over a lifetime. No treatment was given to those with normal BMD.

The natural history of the condition and impact of treatment following fracture risk assessment was represented through a Markov model (online supplemental figure 2). This Markov model consisted of nine health states: untreated osteoporosis, treated osteoporosis, untreated osteopenia, treated osteopenia, normal BMD, no fracture, MOF or HF, post fracture and dead. The ‘population at risk’ state is not a component of the Markov model but was presented to represent the movement of people in the decision tree. A person in the Markov model began in one of the osteopenia, osteoporosis, normal BMD or no fracture health states. Unless individuals were non-adherent to their treatment, they were assumed to not be able to move between ‘treated’ and ‘untreated’ health states. A separate ‘no fracture’ health state was used to capture a proportion of the at-risk population (osteoporosis and osteopenia treated and untreated as well as people with normal BMD) who will not develop osteoporotic fracture in their lifetime.

The model started with 1000 individuals and every year, each individual either remained in the same state or moved to another state based on the defined transition probabilities that were obtained from literature review; ‘death’ was the absorbing state (online supplemental table 1). A 1-year cycle length was assumed, and no half-cycle correction was applied, and the cohort was followed from 40 years old until an age of 100 years or death, whichever was earlier. The model was run separately for MOF and HF; ‘major osteoporotic fracture’ refers to a composite health state consisting of vertebral, shoulder, wrist and HF.

### Model assumptions

There were a few assumptions made during the construct of the model.

- Non-osteoporotic fractures were excluded because other forms of fracture where the fracture risk assessment strategies would not be useful should not be included in the modelling.
- Fracture risk assessment was assumed to occur once at the beginning of the model.
- DXA was assumed to be 100% accurate (ie, have 100% sensitivity and specificity) in diagnosing osteoporosis and osteopenia.
- Treatment decisions were modelled according to categorical DXA T-score thresholds (osteoporosis vs osteopenia) to reflect prevailing UK primary care practice and available data within the intellectual disability population, although fracture risk is continuous in reality.
- Side effects of bisphosphonates were excluded from the model since the risk of osteonecrosis related to bisphosphonate was very low; around 1 in 10 000 to 1 in 100 000 patient-years.^21^
- There is a 100% fracture risk assessment for Strategy 2.
- The post-fracture cost is the same over time as the year-2 cost after fracture, where year 1 is when the fracture occurred.

### Outcomes

Since utilities of people with ID were not available, we assumed that their utilities would be the same as the general population’s. These values were obtained from Janssen and Szende,^22^ who used EuroQoL 5 Dimensions (EQ5D) index values based on UK-specific time trade-off value sets to obtain utility scores. Utility of treated osteoporosis, treated osteopenia, untreated osteoporosis and untreated osteopenia was assumed to be the same as the utility of a person who is fracture-free. The disutility values due to fracture and post fractures were obtained from the International Costs and Utilities Related to Osteoporotic Fractures Study.^23^ Utility values between 0 (death) and 1 (full health) obtained were used to estimate the quality-adjusted life years (QALYs) gained.

### Costs

The cost parameters are found in online supplemental table 1. The unit cost of one DXA was obtained from the NHS Reference Costs^24^ while the unit cost of annual osteoporosis and osteopenia treatment was obtained from the Prescription Cost Analysis.^25^

Direct healthcare resource use and unit costs due to fracture and post fracture (including inpatient stays with operative procedures, accident and emergency attendances, outpatient visits, primary care visits and prescriptions) were obtained from the linked CPRD Aurum and HES databases of the same cohort of people aged 40–79 with ID. Full details of this process are provided in the online supplemental methods 1. All costs were adjusted to 2021/2022 prices using the NHS Cost Inflation Index.^26^

### Base-case analysis

The cost-effectiveness of Strategy 2 (as previously described and applied to the whole population with ID aged 40–79 years), and Strategy 3 (as previously described and applied to the whole population with ID aged 40–79 years) were compared with Strategy 1 (as previously described and applied to 3% of the population with ID aged 40–79 years). Model outcomes were discounted QALYs and direct healthcare costs. The incremental cost-effectiveness ratio (ICER) was calculated as the incremental cost divided by the incremental QALYs and compared between the intervention strategy (ie, Strategy 2 or Strategy 3) and the comparator strategy (ie, Strategy 1). A discount rate of 3.5% was applied to outcomes and costs following the NICE guidelines.^27^ The NICE cost-effectiveness thresholds of £20 000 per additional QALY^28^ and £30 000 per additional QALY^28^ were used to determine the cost-effectiveness of the fracture risk assessment strategies versus Strategy 1. An additional £15 000 per QALY cost-effectiveness threshold^29^ was also used to reflect recent trends in healthcare decision-making. A fracture risk assessment strategy with an ICER below these cost-effectiveness thresholds was considered to be cost-effective. The net monetary benefit (NMB) of the fracture risk assessment strategies was computed across different cost-effectiveness thresholds. A positive incremental NMB would indicate that the fracture risk assessment strategy is cost-effective compared with Strategy 1 at the given cost-effectiveness threshold. For completeness, the cost-effectiveness of Strategy 2 versus Strategy 3 was also compared. All analysis was conducted using R V.4.4.^30^

### Sensitivity analyses

Deterministic sensitivity analysis and probabilistic sensitivity analyses (PSA) were used to assess the impact of the parameter uncertainties on the cost-effectiveness results. For the former, different scenarios such as adherence to osteoporosis and osteopenia treatment, sensitivity and specificity of QFracture and IDFracture were varied from 20% less than the base value to 100% while the costs listed in online supplemental table 1 either halved or doubled from the base value. Fracture risk assessment using QFracture was varied between 1%–5% according to expert opinion. As the fracture risk assessment in the model was over a 10-year period, but participants were followed up over their lifetime to assess future benefits accrued, two sensitivity analyses were also performed based on a 10-year time horizon or a lifetime fracture risk, derived from the 10-year incidence rate using fixed effect meta-analysis. For the PSA, the parameters were sampled 1000 times using beta distribution for probabilities and utilities and gamma distribution for costs. Simulations from the PSA were presented in a cost-effectiveness plane and a cost-effectiveness acceptability curve (CEAC).

### Subgroup analyses

Subgroup analyses stratified by age group and gender were performed to explore heterogeneity in cost-effectiveness across demographic groups. Based on current NICE criteria for determining risk of MOF and HF,^7 10^ we estimated that approximately 1%–5% of the population with ID would be screened at age 40–49 years, approximately 35%–50% at age 50–64 for women and 50–74 years for men, while all women and men with ID would qualify for screening from age 65 and 75 years, respectively. Our estimates originated from the prevalence within the ID population of the factors considered by NICE as risk factors for MOF and HF and on the incidence of MOF and HF in our population.^4^

### Patient and public involvement

Our patient and public involvement representative (JR), who is a co-investigator and the mother of an individual with intellectual disability, helped shape the research question and advised on how best to share the findings, ensuring that communication was clear, realistic and sensitive to patient concerns.

### Role of the funding source

The funder had no role in study design, data collection, data analysis, data interpretation or writing of the report.

## Results

As part of a check to ensure that the model works correctly, the predicted population size following screening at each health state during cycle 1 of the Markov model for both MOF and HF has been presented in online supplemental table 2.

### Base-case analysis

For MOF, Strategy 2 (ICER: −£2568/QALY) was dominant (ie, less costly and more effective, on average) and Strategy 3 (ICER: £1678/QALY) was cost-effective relative to Strategy 1 at the specified cost-effectiveness thresholds of £15 000/QALY to £30 000/QALY threshold (table 1). On average, for MOF, Strategy 2 was £7.23 less costly and generated 0.0028 more QALYs than Strategy 1, and Strategy 3 was £8.81 more costly and generated 0.0052 more QALYs than Strategy 1. Strategies 2 and 3 had positive incremental NMB values relative to Strategy 1, thus indicating that they generate greater economic benefits than Strategy 1.

**Table 1:** Base-case cost-effectiveness analysis of the three fracture risk assessment strategies

	Mean cost	Mean QALY	Incremental cost	Incremental QALY	ICER (£/QALY)	Incremental net monetary benefit at cost-effectiveness threshold of		
						£15 000/QALY	£20 000/QALY	£30 000/QALY
Major osteoporotic fracture								
Strategy 1	2724	12.22	–	–	–	–	–	–
Strategy 2	2717	12.22	−7.23	0.0028	−2568	49	64	92
Strategy 3	2733	12.22	8.81	0.0052	1678	70	96	149
Hip fracture								
Strategy 1	1710	12.27	–	–	–	–	–	–
Strategy 2	1747	12.27	37.76	0.0012	32 116	−20	−14	−2
Strategy 3	1785	12.27	75.68	0.0015	49 536	−53	−45	−30

ICER, incremental cost-effectiveness ratio; QALY, quality-adjusted life year.

For HF, Strategy 2 (ICER: £32 116/QALY) and Strategy 3 (ICER: £49 536/QALY) were not cost-effective relative to Strategy 1 at the £15 000/QALY to £20 000/QALY thresholds (table 1). On average, for HF, Strategy 2 was £37.76 more costly and generated 0.0012 more QALYs than Strategy 1, and Strategy 3 was £75.68 more costly and generated 0.0015 more QALYs than Strategy 1. Strategy 2 and Strategy 3 had negative incremental NMB values at the £15 000/QALY to £30 000/QALY thresholds, thus indicating that it would only be cost-effective relative to Strategy 1.

The comparison between Strategy 2 and Strategy 3 showed that Strategy 3 was cost-effective relative to Strategy 2 (ICER: £6 594/QALY) for MOF (online supplemental table 5) but it was not cost-effective relative to Strategy 2 (ICER: £107 731/QALY) for HF (online supplemental table 6) at the specified cost-effectiveness thresholds.

### Deterministic sensitivity analyses

For MOF (online supplemental table 3), sensitivity analyses with Strategy 1 as comparator were consistent with the base case, except for Strategy 3 at £15 000/QALY threshold, when DXA cost was doubled and a 10-year time horizon was applied, and at £20 000/QALY threshold when a 10-year time horizon was applied.

For HF (online supplemental table 4), sensitivity analyses with Strategy 1 as comparator were largely consistent with the base case. Strategy 2 became cost-effective under certain conditions: at £15 000/QALY with 100% adherence to osteoporosis treatment, 100% IDFracture specificity and a lifetime fracture risk; at £20 000/QALY with 100% adherence to osteopenia treatment, 100% IDFracture specificity, doubled DXA cost, halved post-fracture cost and a lifetime fracture risk; and at £30 000/QALY with 100% adherence to osteoporosis treatment, 99.9% IDFracture sensitivity, 100% IDFracture specificity, halved osteopenia treatment and DXA costs, doubled fracture and post-fracture costs and a lifetime fracture risk. Strategy 3 became cost-effective at £15 000/QALY with a lifetime fracture risk; at £20 000/QALY with halved DXA cost and a lifetime fracture risk; and at £30 000/QALY with 100% adherence to osteoporosis treatment, halved DXA cost and a lifetime fracture risk.

For MOF (online supplemental table 5), Strategy 2 versus Strategy 3 findings were consistent with the base case except when IDFracture sensitivity was 99.9%; DXA cost doubled, and a 10-year time horizon was adopted under all the prespecified cost-effectiveness thresholds. Further exceptions occurred at £15 000/QALY with 100% adherence to osteopenia treatment, 100% IDFracture specificity and halved post-fracture cost.

For HF (online supplemental table 6), Strategy 2 versus Strategy 3 findings were consistent with the base case except when DXA cost was halved under a £30 000/QALY threshold, and a lifetime fracture risk applied under all the prespecified cost-effectiveness thresholds.

### Probabilistic sensitivity analysis

**Figure 1:**
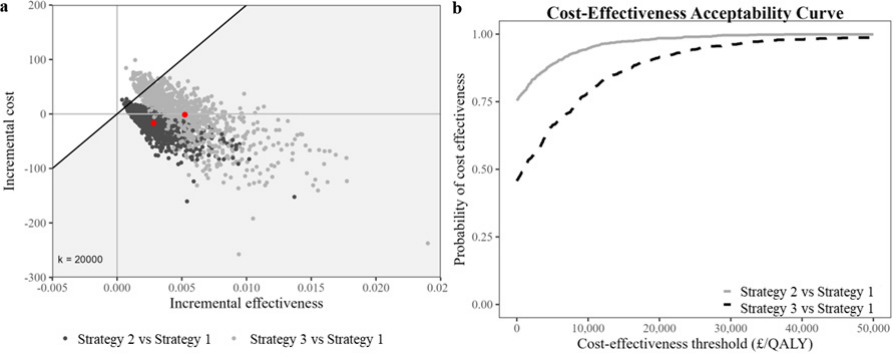
Cost-effectiveness analysis of fracture risk assessment strategies 1–3 among people with intellectual disabilities with major osteoporotic fracture using (**a**) cost-effectiveness plane and (**b**) cost-effectiveness acceptability curve. QALY, quality-adjusted life year.

In the MOF cost-effectiveness plane (figure 1a), PSA scatter points for Strategies 2 and 3 lay mainly below the £20 000/QALY cost-effectiveness threshold. Their CEAC (figure 1b) showed an above 50% probability of cost-effectiveness at the NICE-recommended cost-effectiveness thresholds, suggesting both strategies are cost-effective relative to Strategy 1.

**Figure 2:**
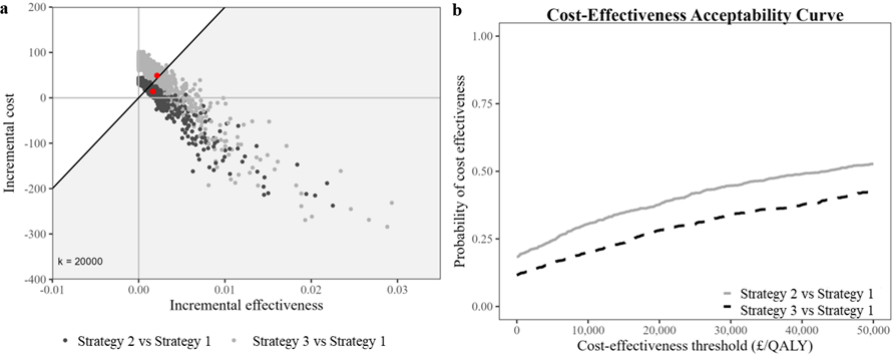
Cost-effectiveness analysis of fracture risk assessment strategies 1–3 among people with intellectual disabilities with hip fracture using (**a**) cost-effectiveness plane and (**b**) cost-effectiveness acceptability curve. QALY, quality-adjusted life year

For HF (figure 2a), PSA scatter points for Strategies 2 and 3 lay mainly above the £20 000/QALY threshold. Their CEAC (figure 2b) showed a below 50% probability, indicating both unlikely to be cost-effective relative to Strategy 1.

When Strategy 3 was compared with Strategy 2, MOF results (online supplemental figure 3a,b) showed an above 50% probability of cost-effectiveness, but HF results (online supplemental figure 4a,b) were below 50%, suggesting Strategy 3 is cost-effective for MOF but not for HF.

### Subgroup analyses

For MOF (online supplemental table 5), subgroup results for Strategies 1 and 2 largely matched the base case, except in men. Strategies 2 and 3 were not cost-effective relative to Strategy 1 among men aged 75–79 years at all thresholds. Strategy 2 also lost cost-effectiveness at £15 000/QALY in people aged 40–48 and men aged 50–74, while Strategy 3 was not cost-effective at £15 000–20 000/QALY in people aged 40–49 and men aged 50–74, and at £30 000/QALY in the same groups at 50% risk assessment. For HF (online supplemental table 5), subgroup results mirrored the base case.

For MOF (online supplemental table 5), comparing Strategies 2 and 3, results mirrored the base case except in men aged 40–79 and women aged 40–49. For HF (online supplemental table 5), comparing Strategies 2 and 3, results also mirrored the base case.

## Discussion

This study showed that for people with ID aged 40–79 years, Strategy 3 (ie, DXA alone) is more cost-effective than Strategy 2 (ie, IDFracture followed by DXA) for MOF, and both would be more cost-effective than the current policy, Strategy 1 (ie, QFracture followed by DXA in selected at risk groups). As HF is a subgroup of MOF, this finding is the most clinically important in terms of policy implication.

Looking in more detail, based on the estimates and assumptions of our health economics model, different strategies could be adopted for different age groups and genders. For MOF, DXA would be the most cost-effective among women with ID, except among those aged 40–49 years where IDFracture, followed by DXA, would be the most cost-effective strategy. IDFracture followed by DXA would be the most cost-effective strategy among men with ID aged 40–74 years and QFracture followed by DXA among men with ID aged 75–79 years for MOF. For HF, QFracture followed by DXA would be the most cost-effective among the subgroups.

However, considering the population as a whole, DXA would be the most cost-effective strategy for MOF, and QFracture followed by DXA would be the most cost-effective strategy for HF among people with ID aged 40 years and above. We were interested in studying HF as an important outcome given its high relative risk, particularly in the younger people with ID, but HF is a subgroup of MOF and the MOF findings are primary in terms of clinical implication.

To the best of our knowledge, this is the first study that evaluated the cost-effectiveness of different fracture risk assessment strategies and certainly the first to do so in the ID community. We accessed a large UK health database linking primary and secondary care records, providing a robust real-world foundation for this health economic analysis. The model we have constructed is different from published osteoporosis models^31^,^33^ because we chose to compare the costs and effects of fracture prevention in the osteoporosis and osteopenia health states separately. Our model compared three strategies, rather than the risk assessment tools that support them (QFracture, IDFracture and DXA), to best inform future policy in addressing this area of healthcare.

However, this study is not without its limitations. First, many parameters are not based on people with ID but we have tried to use intellectual disability-specific estimates whenever available. One such example is the cost of fractures.^11^ Next, we did not include costs from the societal perspective, and this underestimates the additional economic burden of fractures (and the benefits of preventing them) because residential care or supportive living accommodation and individual productivity loss may be cost drivers.^34^ We assumed that all people offered a DXA scan would receive this investigation, but in fact, a proportion of the ID community may not easily tolerate this procedure. Research to investigate more acceptable forms of measurement in this population is a priority. A proportion of people suffering an osteoporotic fracture may have had a previously normal DXA scan, a fact built into our model. In addition, the benefits of fracture risk assessment may have been underestimated, as only individuals diagnosed with osteoporosis on DXA were assumed to receive antiresorptive therapy. We had also assumed treatment over a lifetime due to model constraints, which is not the current practice where the need for continued treatment of bisphosphonate is re-evaluated after 5 years of bisphosphonate treatment.^5^ This would overestimate the cost of osteoporosis treatment. We extrapolated the 10-year fracture risk to a lifetime horizon linearly in our sensitivity analysis, but the fracture risk is likely to increase exponentially over time.^4^ We had used the 2012 version of the QFracture algorithm, so our computation differed slightly from the latest 2016 version, and we did not have all variables used in the algorithm in our data, for example, family history of osteoporosis, living in a nursing or care home, so our QFracture predicted risks were not identical to those that would have been produced by the risk calculator. Lastly, we had assumed a constant post-fracture cost even though it is likely to decrease over time.^35^

## Conclusion

In conclusion, we have shown that current osteoporosis guidelines are not fit for purpose in people with ID, and that there is a pressing need to update them to prevent osteoporotic fractures, devastating for the individual and costly for the health system, in this high-risk population. A diagnosis of ID should be recognised not only as a risk factor (evidenced in previous studies), but also as an opportunity for cost-effective (in fact cost-saving) interventions to prevent fractures in this vulnerable group. This requires a systematic approach for those aged 40–79 years, using either a measurement of BMD or a risk assessment tool tailored to this population.

## Supplementary material

10.1136/bmjopen-2025-110008 online supplemental file 1

## Data Availability

Data may be obtained from a third party and are not publicly available.

## References

[1] Johnell O, Kanis JA (2006). An estimate of the worldwide prevalence and disability associated with osteoporotic fractures. Osteoporos Int.

[2] International Osteoporosis Foundation (2022). Epidemiology (Cambridge, Mass.).

[3] Greendale G, Barrett-Connor E (2001). Outcomes of Osteoporotic Fractures.

[4] Frighi V, Smith M, Andrews TM (2022). Incidence of fractures in people with intellectual disabilities over the life course: a retrospective matched cohort study. EClinicalMedicine.

[5] National Osteoporosis Guideline Group (2021). Clinical guideline for the prevention and treatment of osteoporosis.

[6] Smith M, Roast J, Collins GS Development and external validation of prediction models for major osteoporotic fracture and hip fracture in people with intellectual disability. Osteoporos Int.

[7] National Institute for Health and Care Excellence (2023). Osteoporosis - prevention of fragility fractures.

[8] Husereau D, Drummond M, Augustovski F (2022). Consolidated Health Economic Evaluation Reporting Standards (CHEERS) 2022 Explanation and Elaboration: A Report of the ISPOR CHEERS II Good Practices Task Force. Value Health.

[9] Hippisley-Cox J, Coupland C (2012). Derivation and validation of updated QFracture algorithm to predict risk of osteoporotic fracture in primary care in the United Kingdom: prospective open cohort study. BMJ.

[10] National Institute for Health and Care Excellence (2017). Osteoporosis: assessing the risk of fragility fracture.

[11] National Institute for Health and Care Excellence (2025). Osteoporosis - prevention of fragility fractures: Scenario: Assessment.

[12] Frighi V, Andrews T, Clifton L (2018). Fractures in people with intellectual disabilities: comparison with the general population and development of a fracture risk calculator specific to these patients.

[13] Scottish Intercollegiate Guidelines Network (2021). Management of osteoporosis and the prevention of fragility fractures. SIGN142. https://www.sign.ac.uk/media/1812/sign-142-osteoporosis-v3.pdf.

[14] NHS Digital (2022). Hospital episode statistics (HES). https://digital.nhs.uk/data-and-information/data-tools-and-services/data-services/hospital-episode-statistics.

[15] Clinical Practice Research Datalink (2022). Primary care data for public health research.

[16] Wolf A, Dedman D, Campbell J (2019). Data resource profile: Clinical Practice Research Datalink (CPRD) Aurum. Int J Epidemiol.

[17] Bone Health & Osteoporosis Foundation (2022). Evaluation of Bone Health/Bone Density Testing.

[18] National Osteoporosis Guideline Group (2021). Clinical guideline for the prevention and treatment of osteoporosis.

[19] National Institute for Health and Care Excellence (2017). Bisphosphonates for treating osteoporosis.

[20] Frighi V, Morovat A, Stephenson MT (2014). Vitamin D deficiency in patients with intellectual disabilities: prevalence, risk factors and management strategies. Br J Psychiatry.

[21] Khan AA, Morrison A, Hanley DA (2015). Diagnosis and management of osteonecrosis of the jaw: a systematic review and international consensus. J Bone Miner Res.

[22] Janssen B, Szende A, Szende A, Janssen B, Cabases J (2014). Population Norms for the EQ-5D.

[23] Svedbom A, Borgstöm F, Hernlund E (2018). Quality of life for up to 18 months after low-energy hip, vertebral, and distal forearm fractures-results from the ICUROS. Osteoporos Int.

[24] National Health Service (2024). 2021/22 national cost collection data publication. https://www.england.nhs.uk/publication/2021-22-national-cost-collection-data-publication/.

[25] NHS Business Services Authority (2022). Prescription cost analysis – England – 2021/22. https://www.nhsbsa.nhs.uk/statistical-collections/prescription-cost-analysis-england/prescription-cost-analysis-england-202122.

[26] Jones KC, Weatherly H, Birch S (2023). Unit costs of health and social care 2022.

[27] National Institute for Health and Care Excellence (2022). NICE health technology evaluations: the manual. https://www.nice.org.uk/process/pmg36/chapter/introduction-to-health-technology-evaluation.

[28] National Institute for Health and Care Excellence (2013). Guide to the methods of technology appraisal 2013.

[29] National Institute for Health and Care Excellence (2016). The social care guidance manual. https://www.nice.org.uk/process/pmg10/resources/.

[30] R Core Team (2025). R: A language and environment for statistical computing.

[31] Kanis JA, Adams J, Borgström F (2008). The cost-effectiveness of alendronate in the management of osteoporosis. Bone.

[32] Söreskog E, Borgström F, Lindberg I (2021). A novel economic framework to assess the cost-effectiveness of bone-forming agents in the prevention of fractures in patients with osteoporosis. Osteoporos Int.

[33] Hiligsmann M, Ethgen O, Bruyère O (2009). Development and validation of a Markov microsimulation model for the economic evaluation of treatments in osteoporosis. Value Health.

[34] Schofield D, Zeppel MJB, Tanton R (2019). Intellectual disability and autism: socioeconomic impacts of informal caring, projected to 2030. Br J Psychiatry.

[35] Leslie WD, Lix LM, Finlayson GS (2013). Direct healthcare costs for 5 years post-fracture in Canada: A long-term population-based assessment. Osteoporos Int.

