# Supplementary material for: Cost-effectiveness of osteoporotic fracture risk assessment in people with intellectual disabilities: a UK NHS modelling study
Source: BMJ Open. 2026 Apr 17;16(4):e110008. doi: 10.1136/bmjopen-2025-110008 (PMC13110644; doi:10.1136/bmjopen-2025-110008)
Supplement: online supplemental file 1 [file bmjopen-16-4-s001.docx]

**Cost-effectiveness of osteoporotic fracture risk assessment in people with intellectual disabilities: a UK NHS modelling study**

**SUPPLEMENTARY MATERIAL**

Contents

[SUPPLEMENTARY METHODS 1 2](#_Toc206582342)

[TABLES 3](#_Toc206582343)

[Supplementary Table 1. Model parameters 3](#_Toc206582344)

[Supplementary Table 2. Predicted population size following screening during cycle 1 of the Markov model for major osteoporotic fracture and hip fracture 6](#_Toc206582345)

[Supplementary Table 3. Costs and effects of the fracture risk assessment strategies for major osteoporotic fracture based on sensitivity and subgroup analyses 7](#_Toc206582346)

[Supplementary Table 4. Costs and effects of the fracture risk assessment strategies for hip fracture based on sensitivity and subgroup analyses 12](#_Toc206582347)

[Supplementary Table 5. Cost-effectiveness analysis of Strategy 2 vs. Strategy 3 among people with intellectual disabilities with major osteoporotic fracture 17](#_Toc206582348)

[Supplementary Table 6. Cost-effectiveness analysis of Strategy 2 vs. Strategy 3 among people with intellectual disabilities with hip fracture 20](#_Toc206582349)

[FIGURES 23](#_Toc206582350)

[Supplementary Fig. 1. Decision tree model 23](#_Toc206582351)

[Supplementary Fig. 2. Markov model 24](#_Toc206582352)

[Supplementary Fig. 3. Cost-effectiveness analysis of Strategy 2 vs. Strategy 3 among people with intellectual disabilities with major osteoporotic fracture using (a) cost-effectiveness plane and (b) cost-effectiveness acceptability curve. 25](#_Toc206582353)

[Supplementary Fig. 4. Cost-effectiveness analysis of Strategy 2 vs. Strategy 3 among people with intellectual disabilities with hip fracture using (a) cost-effectiveness plane and (b) cost-effectiveness acceptability curve. 26](#_Toc206582354)

[REFERENCES 27](#_Toc206582355)

# SUPPLEMENTARY METHODS 1

**Measurement of costs from routine database**

An index MOF or HF among this cohort of people with ID was defined using a comprehensive list of primary care (SNOMED CT) terms and codes (for CPRD) and ICD-10 terms and codes (for HES) compiled by three clinicians; further details of this process have been described elsewhere[1]. Unit cost of hospital admission was obtained from the NHS Reference Cost 2021/22[2] based on the clinical specialty, inpatient length of stay (short-stay versus long-stay) and type of admission (elective versus non-elective) after generating the Health Resource Group (HRG) codes from the HRG4+ 2021/22 Reference Cost Grouper at the Finished Consultant Episode (FCE) level[3,4]. Hospital admission costs were analysed at the episode level by summing FCE costs within episodes to generate total costs per inpatient episode. A similar process using this grouper was done to obtain the cost of emergency attendances and outpatient visits. The unit cost of primary care prescriptions was obtained from the estimates provided within the CPRD Aurum. All strategies included a general practitioner (GP) consultation cost. Unit cost of primary care consultations was obtained primarily from the Personal Social Services Research Unit (PSSRU) Unit Costs of Health and Social Care 2022 compendium[5]. All primary care consultations were assumed to last for 50 minutes, 9.22 minutes and 4 minutes for home, surgery and phone consultations respectively based on the average time reported in the PSSRU[5,6]. Staff below NHS Agenda for Change (AfC) pay band four were assumed to work 1618 hours per year[5]. Individuals who had died were assumed to incur zero service utilisation and costs.

Using a complete case analysis, the mean cost due to no fracture, fracture and post-fracture were computed using a two-parts model, which accounted for the skewed distribution of economic costs as a high frequency of patients incurred zero or low costs and a few patients incurred extremely high costs. This model had two stages - a logistic regression, in which the dependent variable (total cost) indicated presence of zero costs; followed by a generalised linear model with a Gaussian distribution and log-link function for economic costs relating to patients with positive values. The unit cost of post-fracture after the first-year post-fracture was assumed to be the same as the first-year post-fracture unit cost. The two-parts model was adjusted using age, gender, Index of Multiple Deprivation score and baseline cost (i.e. healthcare cost incurred within one year before the index fracture).

The adjusted mean total costs of fracture and post-fracture from the NHS perspective was computed from the sum of the adjusted mean costs of inpatient stays including operative procedures, emergency attendances, outpatient visits as well as primary care visits and prescriptions.

# TABLES

## Supplementary Table 1. Model parameters

| **Parameter** | **Value** | **Reference** |
| --- | --- | --- |
| **Transition probability** |  |  |
| Sensitivity of QFracture at 10% cutoff for MOF among people with ID (%) | 17.9 (95%CI 16.2-19.5) | HES-CPRD |
| Sensitivity of QFracture at 3% cutoff for hip fracture among people with ID (%) | 34.3 (95%CI 31.2-37.4) | HES-CPRD |
| Specificity of QFracture at 10% cutoff for MOF among people with ID (%) | 92.9 (9%%CI 92.6-93.1) | HES-CPRD |
| Specificity of QFracture at 3% cutoff for HF among people with ID (%) | 88.5 (95%CI 88.2-88.8) | HES-CPRD |
| Sensitivity of IDFracture at 10% cutoff for MOF among people with ID (%) | 53.9 (95%CI 51.7-56.0) | HES-CPRD |
| Sensitivity of IDFracture at 3% cutoff for HF among people with ID (%) | 77.2 (95%CI 74.4-79.9) | HES-CPRD |
| Specificity of IDFracture at 10% cutoff for MOF among people with ID (%) | 73.7 (95%CI 73.2-74.1) | HES-CPRD |
| Specificity of IDFracture at 3% cutoff for HF among people with ID (%) | 62.7 (95%CI 62.2-63.2) | HES-CPRD |
| Sensitivity of DXA (%) | 100 | assumed |
| Specificity of DXA (%) | 100 | assumed |
| Prevalence of osteopenia per year (%) | 33.2 | [7] |
| Prevalence of osteoporosis per year (%) | 41 | [7] |
| Probability of developing MOF within 10 years among people with ID | Men, 40-79yo: 0.0695 (95%CI 0.0650-0.0743)  Men, 40-49yo: 0.0442 (95%CI 0.0391-0.0500)  Men, 50-74yo: 0.0874 (95%CI 0.0804-0.0951)  Men, 75-79yo: 0.2432 (95%CI 0.1856-0.3149)  Women, 40-79yo: 0.1173 (95%CI 0.1106-0.1243) Women, 40-49yo: 0.0606 (95%CI 0.0534-0.0687)  Women, 50-64yo: 0.1347 (95%CI 0.1236-0.1467)  Women, 65-79yo: 0.2565 (95%CI 0.2308-0.2845) | HES-CPRD |
| Probability of developing HF within 10 years among people with ID | Men, 40-79yo: 0.0352 (95%CI 0.0320-0.0387)  Men, 40-49yo: 0.0177 (95%CI 0.0145-0.0216)  Men, 50-74yo: 0.0476 (95%CI 0.0424-0.0534)  Men, 75-79yo: 0.1612 (95%CI 0.1134-0.2265)  Women, 40-79yo: 0.0456 (95%CI 0.0414-0.0503)  Women, 40-49yo: 0.0127 (95%CI 0.0095-0.0168)  Women, 50-64yo: 0.0490 (95%CI 0.0421-0.0570)  Women, 65-79yo: 0.1509 (95%CI 0.1298-0.1750) | HES-CPRD |
| Probability of developing MOF over a lifetime among people with ID | Men, 40-79yo: 0.1761  Men, 40-49yo: 0.0988  Men, 50-74yo: 0.2014  Men, 75-79yo: 0.4008  Women, 40-79yo: 0.2902  Women, 40-49yo: 0.1003  Women, 50-64yo: 0.2732  Women, 65-79yo: 0.4670 | derived from 10-year probability of developing MOF |
| Probability of developing HF over a lifetime among people with ID | Men, 40-79yo: 0.0997  Men, 40-49yo: 0.0378  Men, 50-74yo: 0.1157  Men, 75-79yo: 0.2476  Women, 40-79yo: 0.1302  Women, 40-49yo: 0.0218  Women, 50-64yo: 0.1009  Women, 65-79yo: 0.2401 | derived from 10-year probability of developing HF |
| Risk assessment (%) | 40-49 yo: 3 (range: 1-5)  Men, 50-74 yo: 35-50  Men, 75-79 yo: 100  Women, 50-64 yo: 35  Women, 65-79 yo: 100 | expert opinion |
| 10-year probability of MOF among people without ID but with a previous fragility fracture (%)* | Men: 9.6  Women: 16.4 | [8] |
| 10-year probability of HF among people without ID but with a previous fragility fracture (%)* | Men: 1.9  Women: 3.2 | [8] |
| Proportion of MOF that has osteoporosis (%) | Men: 20.69  Women: 44.09 | [9] |
| Proportion of HF that has osteoporosis (%) | Men: 38.89  Women: 63.79 | [9] |
| Proportion of MOF that has osteopenia (%) | Men: 61.38  Women: 43.29 | [9] |
| Proportion of HF that has osteopenia (%) | Men: 58.33  Women: 31.03 | [9] |
| Relative risk of MOF after alendronate** | 0.53 (95%CI 0.44-0.65) | [10] |
| Relative risk of HF after alendronate** | 0.61 (95%CI 0.39-0.95) | [10] |
| Relative risk of MOF after calcium and Vitamin D | 0.94 (95%CI 0.89-0.99) | [11] |
| Relative risk of HF after calcium and Vitamin D | 0.84 (95%CI 0.72-0.97) | [11] |
| Hazard ratio of death after MOF | Men: 2.00 (95%CI 1.88-2.12)  Women: 1.37 (95%CI 1.31-1.43) | [12] |
| Hazard ratio of death after HF | Men: 2.13 (95%CI 1.96-2.31)  Women: 1.60 (95%CI 1.50-1.70) | [12] |
| Adherence to osteoporosis treatment (%) | 43 (95%CI 38-49) | [13] |
| Adherence to osteopenia treatment among people with ID (%) | 80.4 | [14] |
| Mortality rate (per 1000 population) among people with ID | Men, 35-44yo: 4.4 (95%CI 2.8-6.7)  Men, 45-54yo: 10.8 (95%CI 8.3-13.9)  Men, 55-64yo: 26.7 (95%CI 21.5-32.8)  Men, 65-74yo: 45.7 (95%CI 36.4-56.8)  Men, 75-84yo: 104.3 (95%CI 79.0-135.2)  Men, 85-99yo: 221.0 (95%CI 136.8-337.9)  Women, 35-44yo: 4.4 (95%CI 2.7-6.8)  Women, 45-54yo: 8.2 (95%CI 5.8-11.1)  Women, 55-64yo: 21.6 (95%CI 16.6-27.6)  Women, 65-74yo: 35.1 (95%CI 26.4-45.6)  Women, 75-84yo: 85.7 (95%CI 63.6-113.0)  Women, 85-99yo: 222.4 (95%CI 147.8-321.5) | [15] |
| **Utility^†^** |  |  |
| Fracture-free | 35-44 yo: 0.893  45-54 yo: 0.855  55-64 yo: 0.810  65-74 yo: 0.773  75+ yo: 0.703 | [16] |
| **Disutility** |  |  |
| MOF | 0.27 (95%CI 0.24-0.29) | [17] |
| Hip fracture | 0.34 (95%CI 0.33-0.36) | [17] |
| Post MOF | 0.13 (95%CI 0.10-0.15) | [17] |
| Post hip fracture | 0.11 (95%CI 0.09-0.12) | [17] |
| **Annual cost (£), in 2021/22 prices** |  |  |
| DXA | 95.45 | [2] |
| General practitioner consultation | 41 | [18] |
| Bisphosphonate (i.e. Alendronate 10mg) | 91.25 | [19] |
| Calcium and vitamin D (i.e. Calcichew 1000mg/800unit) | 83.95 | [19] |
| First year MOF cost among people with ID^‡^ | 9,407 (SE 284) | HES-CPRD |
| First year HF cost among people with ID^‡^ | 12,761 (SE 410) | HES-CPRD |
| Post 1-year MOF cost among people with ID^‡^ | 5,687 (SE 594) | HES-CPRD |
| Post 1-year HF cost among people with ID^‡^ | 5,319 (SE 885) | HES-CPRD |

CI: confidence interval, CPRD: Clinical Practice Research Datalink, DXA: dual-energy x-ray absorptiometry, HES: Hospital Episode Statistics, HF: Hip fracture, HRQoL: health-related quality of life, ID: intellectual disabilities, MOF: major osteoporotic fracture, SE: standard error, yo: years old

*Converted to 1-year probability using the formula: *p’* = 1-(1-p)^1/t^ where *p* is the original probability and *t* is time.

** People with alendronate in this network meta-analysis also had calcium and Vitamin D.

^†^Utility of treated osteoporosis, treated osteopenia, untreated osteoporosis and untreated osteopenia were assumed to be the same as the utility of a person who is fracture-free.

Recovery rate of major osteoporotic fracture was assumed to be the same as hip fracture.

^‡^Adjusted by age, gender, Indices of Multiple Deprivation quintile and baseline cost.

## Supplementary Table 2. Predicted population size following screening during cycle 1 of the Markov model for major osteoporotic fracture and hip fracture

|  | **Untreated osteoporosis** | **Treated osteoporosis** | **Treated osteopenia** | **Untreated osteopenia** | **Normal BMD** | **No Fracture** | **Fracture** | **Post-fracture** | **Dead** |
| --- | --- | --- | --- | --- | --- | --- | --- | --- | --- |
| Major osteoporotic fracture |  |  |  |  |  |  |  |  |  |
| Strategy 1 | 38.08836122 | 0.2056388 | 0.1665173 | 30.84228274 | 24.0972 | 906.6 | 0 | 0 | 0 |
| Strategy 2 | 17.653534 | 20.64047 | 16.71374 | 14.2950568 | 24.0972 | 906.6 | 0 | 0 | 0 |
| Strategy 3 | 0 | 38.294 | 31.0088 | 0 | 24.0972 | 906.6 | 0 | 0 | 0 |
| Hip fracture |  |  |  |  |  |  |  |  |  |
| Strategy 1 | 16.39355644 | 0.1704436 | 0.1380177 | 13.27478229 | 10.4232 | 959.6 | 0 | 0 | 0 |
| Strategy 2 | 3.776592 | 12.78741 | 10.35468 | 3.0581184 | 10.4232 | 959.6 | 0 | 0 | 0 |
| Strategy 3 | 0 | 16.564 | 13.4128 | 0 | 10.4232 | 959.6 | 0 | 0 | 0 |

BMD: bone mineral density, DXA: dual-energy x-ray absorptiometry

## Supplementary Table 3. Costs and effects of the fracture risk assessment strategies for major osteoporotic fracture based on sensitivity and subgroup analyses

|  | **Mean Cost** | **Mean QALY** | **Incremental Cost** | **Incremental QALY** | **ICER (£/QALY)** | **Incremental net monetary benefit at cost-effectiveness threshold of** | | |
| --- | --- | --- | --- | --- | --- | --- | --- | --- |
|  |  |  |  |  |  | **£15 000/ QALY** | **£20 000/ QALY** | **£30 000/ QALY** |
| **Sensitivity analysis** |  |  |  |  |  |  |  |  |
| *Risk assessment at 1% for Strategy 1* |  |  |  |  |  |  |  |  |
| Strategy 1 | 2,724 | 12.22 | - | - | - | - | - | - |
| Strategy 2 | 2,717 | 12.22 | -7.32 | 0.0028 | -2,583 | 50 | 64 | 92 |
| Strategy 3 | 2,733 | 12.22 | 8.72 | 0.0053 | 1,655 | 70 | 97 | 149 |
| *Risk assessment at 5% for Strategy 1* |  |  |  |  |  |  |  |  |
| Strategy 1 | 2,724 | 12.22 | - | - | - | - | - | - |
| Strategy 2 | 2,717 | 12.22 | -7.14 | 0.0028 | -2,554 | 49 | 63 | 91 |
| Strategy 3 | 2,733 | 12.22 | 8.90 | 0.0052 | 1,701 | 70 | 96 | 148 |
| *Adherence to osteoporosis treatment -20%* |  |  |  |  |  |  |  |  |
| Strategy 1 | 2,744 | 12.21 | - | - | - | - | - | - |
| Strategy 2 | 2,737 | 12.22 | -6.62 | 0.0027 | -2,412 | 48 | 62 | 89 |
| Strategy 3 | 2,754 | 12.22 | 9.95 | 0.0051 | 1,946 | 67 | 92 | 143 |
| *Adherence to osteoporosis treatment at 100%* |  |  |  |  |  |  |  |  |
| Strategy 1 | 2,618 | 12.22 | - | - | - | - | - | - |
| Strategy 2 | 2,566 | 12.23 | -52.15 | 0.0074 | -7,007 | 164 | 201 | 275 |
| Strategy 3 | 2,543 | 12.24 | -74.91 | 0.0139 | -5,400 | 283 | 352 | 491 |
| *Adherence to osteopenia treatment -20%* |  |  |  |  |  |  |  |  |
| Strategy 1 | 2,762 | 12.21 | - | - | - | - | - | - |
| Strategy 2 | 2,750 | 12.22 | -11.77 | 0.0029 | -4,019 | 56 | 70 | 100 |
| Strategy 3 | 2,762 | 12.22 | 0.35 | 0.0055 | 63 | 82 | 109 | 163 |
| *Adherence to osteopenia treatment at 100%* |  |  |  |  |  |  |  |  |
| Strategy 1 | 2,684 | 12.22 | - | - | - | - | - | - |
| Strategy 2 | 2,696 | 12.22 | 11.64 | 0.0024 | 4,770 | 25 | 37 | 62 |
| Strategy 3 | 2,728 | 12.22 | 43.98 | 0.0045 | 9,672 | 24 | 47 | 92 |
| *Sensitivity of IDFracture for fracture -20%* |  |  |  |  |  |  |  |  |
| Strategy 1 | 2,724 | 12.22 | - | - | - | - | - | - |
| Strategy 2 | 2,725 | 12.22 | 0.75 | 0.0022 | 332 | 33 | 44 | 67 |
| Strategy 3 | 2,733 | 12.22 | 8.81 | 0.0052 | 1,678 | 70 | 96 | 149 |
| *Sensitivity of IDFracture for fracture at 99.9%** |  |  |  |  |  |  |  |  |
| Strategy 1 | 2,724 | 12.22 | - | - | - | - | - | - |
| Strategy 2 | 2,683 | 12.22 | -41.29 | 0.0052 | -7,873 | 120 | 146 | 199 |
| Strategy 3 | 2,733 | 12.22 | 8.81 | 0.0052 | 1,678 | 70 | 96 | 149 |
| *Specificity of IDFracture for fracture -20%* |  |  |  |  |  |  |  |  |
| Strategy 1 | 2,724 | 12.22 | - | - | - | - | - | - |
| Strategy 2 | 2,735 | 12.22 | 11.00 | 0.0028 | 3,906 | 31 | 45 | 73 |
| Strategy 3 | 2,733 | 12.22 | 8.81 | 0.0052 | 1,678 | 70 | 96 | 149 |
| *Specificity of IDFracture for fracture at 100%* |  |  |  |  |  |  |  |  |
| Strategy 1 | 2,724 | 12.22 | - | - | - | - | - | - |
| Strategy 2 | 2,684 | 12.22 | -39.77 | 0.0028 | -14,120 | 82 | 96 | 124 |
| Strategy 3 | 2,733 | 12.22 | 8.81 | 0.0052 | 1,678 | 70 | 96 | 149 |
| *Sensitivity of QFracture for fracture -20%* |  |  |  |  |  |  |  |  |
| Strategy 1 | 2,724 | 12.22 | - | - | - | - | - | - |
| Strategy 2 | 2,717 | 12.22 | -7.31 | 0.0028 | -2,591 | 50 | 64 | 92 |
| Strategy 3 | 2,733 | 12.22 | 8.73 | 0.0053 | 1,661 | 70 | 96 | 149 |
| *Sensitivity of QFracture for fracture at 100%* |  |  |  |  |  |  |  |  |
| Strategy 1 | 2,722 | 12.22 | - | - | - | - | - | - |
| Strategy 2 | 2,717 | 12.22 | -5.41 | 0.0027 | -2,014 | 46 | 59 | 86 |
| Strategy 3 | 2,733 | 12.22 | 10.63 | 0.0051 | 2,077 | 66 | 92 | 143 |
| *Specificity of QFracture for fracture -20%* |  |  |  |  |  |  |  |  |
| Strategy 1 | 2,725 | 12.22 | - | - | - | - | - | - |
| Strategy 2 | 2,717 | 12.22 | -7.92 | 0.0028 | -2,813 | 50 | 64 | 92 |
| Strategy 3 | 2,733 | 12.22 | 8.12 | 0.0052 | 1,547 | 71 | 97 | 149 |
| *Specificity of QFracture for fracture at 100%* |  |  |  |  |  |  |  |  |
| Strategy 1 | 2,724 | 12.22 | - | - | - | - | - | - |
| Strategy 2 | 2,717 | 12.22 | -6.97 | 0.0028 | -2,475 | 49 | 63 | 91 |
| Strategy 3 | 2,733 | 12.22 | 9.07 | 0.0052 | 1,728 | 70 | 96 | 148 |
| *Cost of alendronate -50%* |  |  |  |  |  |  |  |  |
| Strategy 1 | 2,479 | 12.22 | - | - | - | - | - | - |
| Strategy 2 | 2,471 | 12.22 | -8.73 | 0.0028 | -3,101 | 51 | 65 | 93 |
| Strategy 3 | 2,485 | 12.22 | 6.01 | 0.0052 | 1,146 | 73 | 99 | 151 |
| *Cost of alendronate +100%* |  |  |  |  |  |  |  |  |
| Strategy 1 | 3,213 | 12.22 | - | - | - | - | - | - |
| Strategy 2 | 3,209 | 12.22 | -4.23 | 0.0028 | -1,503 | 46 | 61 | 89 |
| Strategy 3 | 3,228 | 12.22 | 14.40 | 0.0052 | 2,743 | 64 | 91 | 143 |
| *Cost of vitamin D and calcium -50%* |  |  |  |  |  |  |  |  |
| Strategy 1 | 2,317 | 12.22 | - | - | - | - | - | - |
| Strategy 2 | 2,306 | 12.22 | -11.22 | 0.0028 | -3,983 | 53 | 68 | 96 |
| Strategy 3 | 2,318 | 12.22 | 1.38 | 0.0052 | 263 | 77 | 104 | 156 |
| *Cost of vitamin D and calcium +100%* |  |  |  |  |  |  |  |  |
| Strategy 1 | 3,539 | 12.22 | - | - | - | - | - | - |
| Strategy 2 | 3,539 | 12.22 | 0.74 | 0.0028 | 261 | 42 | 56 | 84 |
| Strategy 3 | 3,562 | 12.22 | 23.66 | 0.0052 | 4,508 | 55 | 81 | 134 |
| *Cost of DXA -50%* |  |  |  |  |  |  |  |  |
| Strategy 1 | 2,724 | 12.22 | - | - | - | - | - | - |
| Strategy 2 | 2,703 | 12.22 | -20.90 | 0.0028 | -7,421 | 63 | 77 | 105 |
| Strategy 3 | 2,685 | 12.22 | -38.80 | 0.0052 | -7,391 | 118 | 144 | 196 |
| *Cost of DXA +100%* |  |  |  |  |  |  |  |  |
| Strategy 1 | 2,724 | 12.22 | - | - | - | - | - | - |
| Strategy 2 | 2,744 | 12.22 | 20.10 | 0.0028 | 7,136 | 22 | 36 | 64 |
| Strategy 3 | 2,828 | 12.22 | 104.03 | 0.0052 | 19,817 | -25 | 1 | 53 |
| *Cost of fracture -50%* |  |  |  |  |  |  |  |  |
| Strategy 1 | 2,600 | 12.22 | - | - | - | - | - | - |
| Strategy 2 | 2,597 | 12.22 | -3.14 | 0.0028 | -1,116 | 45 | 59 | 88 |
| Strategy 3 | 2,616 | 12.22 | 16.43 | 0.0052 | 3,130 | 62 | 89 | 141 |
| *Cost of fracture +100%* |  |  |  |  |  |  |  |  |
| Strategy 1 | 2,973 | 12.22 | - | - | - | - | - | - |
| Strategy 2 | 2,957 | 12.22 | -15.41 | 0.0028 | -5,473 | 58 | 72 | 100 |
| Strategy 3 | 2,966 | 12.22 | -6.44 | 0.0052 | -1,226 | 85 | 111 | 164 |
| *Cost of post-fracture -50%* |  |  |  |  |  |  |  |  |
| Strategy 1 | 2,159 | 12.22 | - | - | - | - | - | - |
| Strategy 2 | 2,176 | 12.22 | 17.31 | 0.0028 | 6,147 | 25 | 39 | 67 |
| Strategy 3 | 2,214 | 12.22 | 54.56 | 0.0052 | 10,394 | 24 | 50 | 103 |
| *Cost of post-fracture +100%* |  |  |  |  |  |  |  |  |
| Strategy 1 | 3,854 | 12.22 | - | - | - | - | - | - |
| Strategy 2 | 3,798 | 12.22 | -56.33 | 0.0028 | -20,000 | 99 | 113 | 141 |
| Strategy 3 | 3,772 | 12.22 | -82.70 | 0.0052 | -15,754 | 161 | 188 | 240 |
| *10-year time horizon* |  |  |  |  |  |  |  |  |
| Strategy 1 | 1,146 | 7.17 | - | - | - | - | - | - |
| Strategy 2 | 1,160 | 7.17 | 13.78 | 0.0011 | 12,308 | 3 | 9 | 20 |
| Strategy 3 | 1,194 | 7.18 | 47.98 | 0.0021 | 22,990 | -17 | -6 | 15 |
| *Lifetime fracture risk* |  |  |  |  |  |  |  |  |
| Strategy 1 | 7,849 | 11.94 | - | - | - | - | - | - |
| Strategy 2 | 7,668 | 11.95 | -180.87 | 0.0120 | -15,047 | 361 | 421 | 541 |
| Strategy 3 | 7,525 | 11.96 | -324.58 | 0.0224 | -14,488 | 661 | 773 | 997 |
| **Subgroup analysis** |  |  |  |  |  |  |  |  |
| *40-49 years old* |  |  |  |  |  |  |  |  |
| Strategy 1 | 2,181 | 14.12 | - | - | - | - | - | - |
| Strategy 2 | 2,202 | 14.12 | 20.52 | 0.0010 | 19,603 | -5 | 0 | 11 |
| Strategy 3 | 2,245 | 14.13 | 63.40 | 0.0020 | 32,496 | -34 | -24 | -5 |
| *Men aged 50-74 years old, risk assessment at 35% for Strategy 1* |  |  |  |  |  |  |  |  |
| Strategy 1 | 1,568 | 9.74 | - | - | - | - | - | - |
| Strategy 2 | 1,592 | 9.74 | 23.70 | 0.0012 | 19,763 | -6 | 0 | 12 |
| Strategy 3 | 1,638 | 9.74 | 69.95 | 0.0024 | 29,643 | -35 | -23 | 1 |
| *Women aged 50-64 years old, risk assessment at 35% for Strategy 1* |  |  |  |  |  |  |  |  |
| Strategy 1 | 3,137 | 10.87 | - | - | - | - | - | - |
| Strategy 2 | 3,104 | 10.88 | -32.28 | 0.0030 | -10,708 | 77 | 93 | 123 |
| Strategy 3 | 3,093 | 10.88 | -43.51 | 0.0059 | -7,335 | 132 | 162 | 221 |
| *Men aged 50-74 years old, risk assessment at 50% for Strategy 1* |  |  |  |  |  |  |  |  |
| Strategy 1 | 1,569 | 9.74 | - | - | - | - | - | - |
| Strategy 2 | 1,592 | 9.74 | 22.72 | 0.0011 | 20,075 | -6 | 0 | 11 |
| Strategy 3 | 1,638 | 9.74 | 68.96 | 0.0023 | 30,088 | -35 | -23 | -0.2 |
| *Women aged 50-64 years old, risk assessment at 50% for Strategy 1* |  |  |  |  |  |  |  |  |
| Strategy 1 | 3,134 | 10.87 | - | - | - | - | - | - |
| Strategy 2 | 3,104 | 10.88 | -30.13 | 0.0028 | -10,591 | 73 | 87 | 115 |
| Strategy 3 | 3,093 | 10.88 | -41.36 | 0.0058 | -7,178 | 128 | 157 | 214 |
| *Men aged 75-79 years old, risk assessment at 100% for Strategy 1* |  |  |  |  |  |  |  |  |
| Strategy 1 | 773 | 5.07 | - | - | - | - | - | - |
| Strategy 2 | 799 | 5.07 | 25.94 | 0.0005 | 52,222 | -18 | -16 | -11 |
| Strategy 3 | 854 | 5.07 | 80.52 | 0.0011 | 71,074 | -64 | -58 | -47 |
| *Women aged 65-79 years old, risk assessment at 100% for Strategy 1* |  |  |  |  |  |  |  |  |
| Strategy 1 | 1,691 | 6.95 | - | - | - | - | - | - |
| Strategy 2 | 1,688 | 6.96 | -2.59 | 0.0017 | -1,514 | 28 | 37 | 54 |
| Strategy 3 | 1,703 | 6.96 | 12.24 | 0.0039 | 3,135 | 46 | 66 | 105 |

DXA: dual x-ray absorptiometry, ICER: incremental cost-effectiveness ratio, QALY: quality-adjusted life-year

*99.9% instead of 100% was used as the high value for IDFracture sensitivity due to the incremental QALY between Strategy 2 and Strategy 3 to be less than <0.00001 when sensitivity of IDFracture is 100% and the ICER goes to infinity due to the small incremental QALY.

## Supplementary Table 4. Costs and effects of the fracture risk assessment strategies for hip fracture based on sensitivity and subgroup analyses

|  | **Mean Cost** | **Mean QALY** | **Incremental Cost** | **Incremental QALY** | **ICER (£/QALY)** | **Incremental net monetary benefit at cost-effectiveness threshold of** | | |
| --- | --- | --- | --- | --- | --- | --- | --- | --- |
|  |  |  |  |  |  | **£15 000/ QALY** | **£20 000/ QALY** | **£30 000/ QALY** |
| **Sensitivity analysis** |  |  |  |  |  |  |  |  |
| *Risk assessment at 1% for Strategy 1* |  |  |  |  |  |  |  |  |
| Strategy 1 | 1,709 | 12.27 | - | - | - | - | - | - |
| Strategy 2 | 1,747 | 12.27 | 37.97 | 0.0012 | 32,003 | -20 | -14 | -2 |
| Strategy 3 | 1,785 | 12.27 | 75.89 | 0.0015 | 49,328 | -53 | -45 | -30 |
| *Risk assessment at 5% for Strategy 1* |  |  |  |  |  |  |  |  |
| Strategy 1 | 1,710 | 12.27 | - | - | - | - | - | - |
| Strategy 2 | 1,747 | 12.27 | 37.56 | 0.0012 | 32,232 | -20 | -14 | -3 |
| Strategy 3 | 1,785 | 12.27 | 75.48 | 0.0015 | 49,746 | -53 | -45 | -30 |
| *Adherence to osteoporosis treatment -20%* |  |  |  |  |  |  |  |  |
| Strategy 1 | 1,716 | 12.27 | - | - | - | - | - | - |
| Strategy 2 | 1,754 | 12.27 | 37.60 | 0.0012 | 32,467 | -20 | -14 | -3 |
| Strategy 3 | 1,792 | 12.27 | 75.47 | 0.0015 | 50,154 | -53 | -45 | -30 |
| *Adherence to osteoporosis treatment at 100%* |  |  |  |  |  |  |  |  |
| Strategy 1 | 1,675 | 12.27 | - | - | - | - | - | - |
| Strategy 2 | 1,712 | 12.28 | 37.15 | 0.0028 | 13,491 | 4 | 18 | 45 |
| Strategy 3 | 1,750 | 12.28 | 74.88 | 0.0036 | 20,930 | -21 | -3 | 32 |
| *Adherence to osteopenia treatment -20%* |  |  |  |  |  |  |  |  |
| Strategy 1 | 1,717 | 12.27 | - | - | - | - | - | - |
| Strategy 2 | 1,754 | 12.27 | 36.66 | 0.0012 | 31,615 | -19 | -13 | -2 |
| Strategy 3 | 1,791 | 12.27 | 74.25 | 0.0015 | 49,277 | -52 | -44 | -29 |
| *Adherence to osteopenia treatment at 100%* |  |  |  |  |  |  |  |  |
| Strategy 1 | 1,702 | 12.27 | - | - | - | - | - | - |
| Strategy 2 | 1,745 | 12.27 | 43.51 | 0.0013 | 32,804 | -24 | -17 | -4 |
| Strategy 3 | 1,785 | 12.27 | 83.14 | 0.0017 | 48,248 | -57 | -49 | -31 |
| *Sensitivity of IDFracture for fracture -20%* |  |  |  |  |  |  |  |  |
| Strategy 1 | 1,710 | 12.27 | - | - | - | - | - | - |
| Strategy 2 | 1,749 | 12.27 | 39.92 | 0.0009 | 42,578 | -26 | -21 | -12 |
| Strategy 3 | 1,785 | 12.27 | 75.68 | 0.0015 | 49,536 | -53 | -45 | -30 |
| *Sensitivity of IDFracture for fracture at 99.9%** |  |  |  |  |  |  |  |  |
| Strategy 1 | 1,710 | 12.27 | - | - | - | - | - | - |
| Strategy 2 | 1,744 | 12.27 | 34.60 | 0.0015 | 22,668 | -12 | -4 | 11 |
| Strategy 3 | 1,785 | 12.27 | 75.68 | 0.0015 | 49,536 | -53 | -45 | -30 |
| *Specificity of IDFracture for fracture -20%* |  |  |  |  |  |  |  |  |
| Strategy 1 | 1,710 | 12.27 | - | - | - | - | - | - |
| Strategy 2 | 1,764 | 12.27 | 54.18 | 0.0012 | 46,080 | -37 | -31 | -19 |
| Strategy 3 | 1,785 | 12.27 | 75.68 | 0.0015 | 49,536 | -53 | -45 | -30 |
| *Specificity of IDFracture for fracture at 100%* |  |  |  |  |  |  |  |  |
| Strategy 1 | 1,710 | 12.27 | - | - | - | - | - | - |
| Strategy 2 | 1,699 | 12.27 | -11.08 | 0.0012 | -9,419 | 29 | 35 | 46 |
| Strategy 3 | 1,785 | 12.27 | 75.68 | 0.0015 | 49,536 | -53 | -45 | -30 |
| *Sensitivity of QFracture for fracture -20%* |  |  |  |  |  |  |  |  |
| Strategy 1 | 1,710 | 12.27 | - | - | - | - | - | - |
| Strategy 2 | 1,747 | 12.27 | 37.74 | 0.0012 | 32,005 | -20 | -14 | -2 |
| Strategy 3 | 1,785 | 12.27 | 75.65 | 0.0015 | 49,414 | -53 | -45 | -30 |
| *Sensitivity of QFracture for fracture at 100%* |  |  |  |  |  |  |  |  |
| Strategy 1 | 1,709 | 12.27 | - | - | - | - | - | - |
| Strategy 2 | 1,747 | 12.27 | 38.04 | 0.0011 | 33,209 | -21 | -15 | -4 |
| Strategy 3 | 1,785 | 12.27 | 75.96 | 0.0015 | 50,726 | -53 | -46 | -31 |
| *Specificity of QFracture for fracture -20%* |  |  |  |  |  |  |  |  |
| Strategy 1 | 1,710 | 12.27 | - | - | - | - | - | - |
| Strategy 2 | 1,747 | 12.27 | 37.07 | 0.0012 | 31,525 | -19 | -14 | -2 |
| Strategy 3 | 1,785 | 12.27 | 74.99 | 0.0015 | 49,080 | -52 | -44 | -29 |
| *Specificity of QFracture for fracture at 100%* |  |  |  |  |  |  |  |  |
| Strategy 1 | 1,709 | 12.27 | - | - | - | - | - | - |
| Strategy 2 | 1,747 | 12.27 | 38.22 | 0.0012 | 32,500 | -21 | -15 | -3 |
| Strategy 3 | 1,785 | 12.27 | 76.13 | 0.0015 | 49,831 | -53 | -46 | -30 |
| *Cost of alendronate -50%* |  |  |  |  |  |  |  |  |
| Strategy 1 | 1,451 | 12.27 | - | - | - | - | - | - |
| Strategy 2 | 1,487 | 12.27 | 36.84 | 0.0012 | 31,326 | -19 | -13 | -2 |
| Strategy 3 | 1,525 | 12.27 | 74.47 | 0.0015 | 48,746 | -52 | -44 | -29 |
| *Cost of alendronate +100%* |  |  |  |  |  |  |  |  |
| Strategy 1 | 2,227 | 12.27 | - | - | - | - | - | - |
| Strategy 2 | 2,267 | 12.27 | 39.62 | 0.0012 | 33,696 | -22 | -16 | -4 |
| Strategy 3 | 2,306 | 12.27 | 78.10 | 0.0015 | 51,115 | -55 | -48 | -32 |
| *Cost of vitamin D and calcium -50%* |  |  |  |  |  |  |  |  |
| Strategy 1 | 1,278 | 12.27 | - | - | - | - | - | - |
| Strategy 2 | 1,314 | 12.27 | 35.22 | 0.0012 | 29,953 | -18 | -12 | 0.1 |
| Strategy 3 | 1,351 | 12.27 | 72.38 | 0.0015 | 47,373 | -49 | -42 | -27 |
| *Cost of vitamin D and calcium +100%* |  |  |  |  |  |  |  |  |
| Strategy 1 | 2,572 | 12.27 | - | - | - | - | - | - |
| Strategy 2 | 2,615 | 12.27 | 42.85 | 0.0012 | 36,441 | -25 | -19 | -8 |
| Strategy 3 | 2,654 | 12.27 | 82.29 | 0.0015 | 53,861 | -59 | -52 | -36 |
| *Cost of DXA -50%* |  |  |  |  |  |  |  |  |
| Strategy 1 | 1,709 | 12.27 | - | - | - | - | - | - |
| Strategy 2 | 1,729 | 12.27 | 19.37 | 0.0012 | 16,474 | -2 | 4 | 16 |
| Strategy 3 | 1,738 | 12.27 | 28.13 | 0.0015 | 18,415 | -5 | 2 | 18 |
| *Cost of DXA +100%* |  |  |  |  |  |  |  |  |
| Strategy 1 | 1,710 | 12.27 | - | - | - | - | - | - |
| Strategy 2 | 1,784 | 12.27 | 74.55 | 0.0012 | 63,400 | -57 | -51 | -39 |
| Strategy 3 | 1,881 | 12.27 | 170.78 | 0.0015 | 111,777 | -148 | -140 | -125 |
| *Cost of fracture -50%* |  |  |  |  |  |  |  |  |
| Strategy 1 | 1,675 | 12.27 | - | - | - | - | - | - |
| Strategy 2 | 1,715 | 12.27 | 39.81 | 0.0012 | 33,853 | -22 | -16 | -5 |
| Strategy 3 | 1,754 | 12.27 | 78.33 | 0.0015 | 51,272 | -55 | -48 | -33 |
| *Cost of fracture +100%* |  |  |  |  |  |  |  |  |
| Strategy 1 | 1,778 | 12.27 | - | - | - | - | - | - |
| Strategy 2 | 1,812 | 12.27 | 33.68 | 0.0012 | 28,643 | -16 | -10 | 2 |
| Strategy 3 | 1,848 | 12.27 | 70.37 | 0.0015 | 46,062 | -47 | -40 | -25 |
| *Cost of post-fracture -50%* |  |  |  |  |  |  |  |  |
| Strategy 1 | 1,600 | 12.27 | - | - | - | - | - | - |
| Strategy 2 | 1,646 | 12.27 | 46.61 | 0.0012 | 39,635 | -29 | -23 | -11 |
| Strategy 3 | 1,687 | 12.27 | 87.17 | 0.0015 | 57,054 | -64 | -57 | -41 |
| *Cost of post-fracture +100%* |  |  |  |  |  |  |  |  |
| Strategy 1 | 1,929 | 12.27 | - | - | - | - | - | - |
| Strategy 2 | 1,949 | 12.27 | 20.08 | 0.0012 | 17,078 | -2 | 3 | 15 |
| Strategy 3 | 1,982 | 12.27 | 52.71 | 0.0015 | 34,498 | -30 | -22 | -7 |
| *10-year time horizon* |  |  |  |  |  |  |  |  |
| Strategy 1 | 868 | 7.18 | - | - | - | - | - | - |
| Strategy 2 | 914 | 7.18 | 45.61 | 0.0004 | 108,412 | -39 | -37 | -33 |
| Strategy 3 | 954 | 7.18 | 85.88 | 0.0005 | 157,098 | -78 | -75 | -69 |
| *Lifetime fracture risk* |  |  |  |  |  |  |  |  |
| Strategy 1 | 3,240 | 12.18 | - | - | - | - | - | - |
| Strategy 2 | 3,179 | 12.19 | -60.76 | 0.0072 | -8,403 | 169 | 205 | 278 |
| Strategy 3 | 3,183 | 12.19 | -57.59 | 0.0094 | -6,130 | 199 | 246 | 339 |
| **Subgroup analysis** |  |  |  |  |  |  |  |  |
| *40-49 years old* |  |  |  |  |  |  |  |  |
| Strategy 1 | 1,697 | 14.15 | - | - | - | - | - | - |
| Strategy 2 | 1,747 | 14.15 | 49.97 | 0.0002 | 259,532 | -47 | -46 | -44 |
| Strategy 3 | 1,790 | 14.15 | 93.32 | 0.0003 | 373,010 | -90 | -88 | -86 |
| *Men aged 50-74 years old, risk assessment at 35% for Strategy 1* |  |  |  |  |  |  |  |  |
| Strategy 1 | 1,290 | 9.76 | - | - | - | - | - | - |
| Strategy 2 | 1,332 | 9.76 | 41.78 | 0.0007 | 59,658 | -31 | -28 | -21 |
| Strategy 3 | 1,373 | 9.76 | 82.61 | 0.0009 | 87,400 | -68 | -64 | -54 |
| *Women aged 50-64 years old, risk assessment at 35% for Strategy 1* |  |  |  |  |  |  |  |  |
| Strategy 1 | 1,620 | 10.93 | - | - | - | - | - | - |
| Strategy 2 | 1,651 | 10.94 | 31.07 | 0.0010 | 30,617 | -16 | -11 | -1 |
| Strategy 3 | 1,688 | 10.94 | 67.44 | 0.0014 | 49,231 | -47 | -40 | -26 |
| *Men aged 50-74 years old, risk assessment at 50% for Strategy 1* |  |  |  |  |  |  |  |  |
| Strategy 1 | 1,292 | 9.76 | - | - | - | - | - | - |
| Strategy 2 | 1,332 | 9.76 | 39.67 | 0.0006 | 61,497 | -30 | -27 | -20 |
| Strategy 3 | 1,373 | 9.76 | 80.50 | 0.0009 | 90,456 | -67 | -63 | -54 |
| *Women aged 50-64 years old, risk assessment at 50% for Strategy 1* |  |  |  |  |  |  |  |  |
| Strategy 1 | 1,621 | 10.93 | - | - | - | - | - | - |
| Strategy 2 | 1,651 | 10.94 | 29.79 | 0.0009 | 31,872 | -16 | -11 | -2 |
| Strategy 3 | 1,688 | 10.94 | 66.16 | 0.0013 | 51,297 | -47 | -40 | -27 |
| *Men aged 75-79 years old, risk assessment at 100% for Strategy 1* |  |  |  |  |  |  |  |  |
| Strategy 1 | 701 | 5.08 | - | - | - | - | - | - |
| Strategy 2 | 736 | 5.08 | 35.52 | 0.0003 | 140,028 | -32 | -30 | -28 |
| Strategy 3 | 779 | 5.08 | 77.89 | 0.0004 | 200,494 | -72 | -70 | -66 |
| *Women aged 65-79 years old, risk assessment at 100% for Strategy 1* |  |  |  |  |  |  |  |  |
| Strategy 1 | 1,023 | 6.99 | - | - | - | - | - | - |
| Strategy 2 | 1,054 | 6.99 | 30.66 | 0.0005 | 61,866 | -23 | -21 | -16 |
| Strategy 3 | 1,093 | 6.99 | 69.76 | 0.0008 | 91,896 | -58 | -55 | -47 |

DXA: dual x-ray absorptiometry, ICER: incremental cost-effectiveness ratio, QALY: quality-adjusted life-year

*99.9% instead of 100% was used as the high value for IDFracture sensitivity due to the incremental QALY between Strategy 2 and Strategy 3 to be less than <0.00001 when sensitivity of IDFracture is 100% and the ICER goes to infinity due to the small incremental QALY.

## Supplementary Table 5. Cost-effectiveness analysis of Strategy 2 vs. Strategy 3 among people with intellectual disabilities with major osteoporotic fracture

|  | **Mean Cost** | **Mean QALY** | **Incremental Cost** | **Incremental QALY** | **ICER (£/QALY)** | **Incremental net monetary benefit at cost-effectiveness threshold of** | | |
| --- | --- | --- | --- | --- | --- | --- | --- | --- |
|  |  |  |  |  |  | **£15 000/ QALY** | **£20 000/ QALY** | **£30 000/ QALY** |
| **Base case** |  |  |  |  |  |  |  |  |
| Strategy 2 | 2,717 | 12.22 | - | - | - | - | - | - |
| Strategy 3 | 2,733 | 12.22 | 16.04 | 0.0024 | 6,594 | 20 | 33 | 57 |
| **Sensitivity analysis** |  |  |  |  |  |  |  |  |
| *Adherence to osteoporosis treatment -20%* |  |  |  |  |  |  |  |  |
| Strategy 2 | 2,737 | 12.22 | - | - | - | - | - | - |
| Strategy 3 | 2,754 | 12.22 | 16.57 | 0.0024 | 6,991 | 19 | 31 | 55 |
| *Adherence to osteoporosis treatment at 100%* |  |  |  |  |  |  |  |  |
| Strategy 2 | 2,566 | 12.23 | - | - | - | - | - | - |
| Strategy 3 | 2,543 | 12.24 | -22.76 | 0.0064 | -3,540 | 119 | 151 | 216 |
| *Adherence to osteopenia treatment -20%* |  |  |  |  |  |  |  |  |
| Strategy 2 | 2,750 | 12.22 | - | - | - | - | - | - |
| Strategy 3 | 2,762 | 12.22 | 12.12 | 0.0025 | 4,789 | 26 | 39 | 64 |
| *Adherence to osteopenia treatment at 100%* |  |  |  |  |  |  |  |  |
| Strategy 2 | 2,696 | 12.22 | - | - | - | - | - | - |
| Strategy 3 | 2,728 | 12.22 | 32.35 | 0.0021 | 15,347 | -1 | 10 | 31 |
| *Sensitivity of IDFracture for fracture -20%* |  |  |  |  |  |  |  |  |
| Strategy 2 | 2,725 | 12.22 | - | - | - | - | - | - |
| Strategy 3 | 2,733 | 12.22 | 8.06 | 0.0030 | 2,686 | 37 | 52 | 82 |
| *Sensitivity of IDFracture for fracture at 99.9%** |  |  |  |  |  |  |  |  |
| Strategy 2 | 2,683 | 12.22 | - | - | - | - | - | - |
| Strategy 3 | 2,733 | 12.22 | 50.10 | <0.0001 | 9,491,917 | -50 | -50 | -50 |
| *Specificity of IDFracture for fracture -20%* |  |  |  |  |  |  |  |  |
| Strategy 2 | 2,735 | 12.22 | - | - | - | - | - | - |
| Strategy 3 | 2,733 | 12.22 | -2.19 | 0.0024 | -901 | 39 | 51 | 75 |
| *Specificity of IDFracture for fracture at 100%* |  |  |  |  |  |  |  |  |
| Strategy 2 | 2,684 | 12.22 | - | - | - | - | - | - |
| Strategy 3 | 2,733 | 12.22 | 48.58 | 0.0024 | 19,965 | -12 | 0 | 24 |
| *Cost of alendrontate -50%* |  |  |  |  |  |  |  |  |
| Strategy 2 | 2,471 | 12.22 | - | - | - | - | - | - |
| Strategy 3 | 2,485 | 12.22 | 14.75 | 0.0024 | 6,061 | 22 | 34 | 58 |
| *Cost of alendrontate +100%* |  |  |  |  |  |  |  |  |
| Strategy 2 | 3,209 | 12.22 | - | - | - | - | - | - |
| Strategy 3 | 3,228 | 12.22 | 18.64 | 0.0024 | 7,659 | 18 | 30 | 54 |
| *Cost of vitamin D and calcium treatment -50%* |  |  |  |  |  |  |  |  |
| Strategy 2 | 2,306 | 12.22 | - | - | - | - | - | - |
| Strategy 3 | 2,318 | 12.22 | 12.60 | 0.0024 | 5,179 | 24 | 36 | 60 |
| *Cost of vitamin D and calcium treatment +100%* |  |  |  |  |  |  |  |  |
| Strategy 2 | 3,539 | 12.22 | - | - | - | - | - | - |
| Strategy 3 | 3,562 | 12.22 | 22.93 | 0.0024 | 9,423 | 14 | 26 | 50 |
| *Cost of DXA -50%* |  |  |  |  |  |  |  |  |
| Strategy 2 | 2,703 | 12.22 | - | - | - | - | - | - |
| Strategy 3 | 2,685 | 12.22 | -17.90 | 0.0024 | -7,357 | 54 | 67 | 91 |
| *Cost of DXA +100%* |  |  |  |  |  |  |  |  |
| Strategy 2 | 2,744 | 12.22 | - | - | - | - | - | - |
| Strategy 3 | 2,828 | 12.22 | 83.93 | 0.0024 | 34,495 | -47 | -35 | -11 |
| *Cost of fracture -50%* |  |  |  |  |  |  |  |  |
| Strategy 2 | 2,597 | 12.22 | - | - | - | - | - | - |
| Strategy 3 | 2,616 | 12.22 | 19.58 | 0.0024 | 8,046 | 17 | 29 | 53 |
| *Cost of fracture +100%* |  |  |  |  |  |  |  |  |
| Strategy 2 | 2,957 | 12.22 | - | - | - | - | - | - |
| Strategy 3 | 2,966 | 12.22 | 8.98 | 0.0024 | 3,689 | 28 | 40 | 64 |
| *Cost of post-fracture -50%* |  |  |  |  |  |  |  |  |
| Strategy 2 | 2,176 | 12.22 | - | - | - | - | - | - |
| Strategy 3 | 2,214 | 12.22 | 37.25 | 0.0024 | 15,310 | -1 | 11 | 36 |
| *Cost of post-fracture +100%* |  |  |  |  |  |  |  |  |
| Strategy 2 | 3,798 | 12.22 | - | - | - | - | - | - |
| Strategy 3 | 3,772 | 12.22 | -26.37 | 0.0024 | -10,838 | 63 | 75 | 99 |
| *10-year time horizon* |  |  |  |  |  |  |  |  |
| Strategy 2 | 1,160 | 7.17 | - | - | - | - | - | - |
| Strategy 3 | 1,194 | 7.18 | 34.20 | 0.0010 | 35,355 | -20 | -15 | -5 |
| *Lifetime fracture risk* |  |  |  |  |  |  |  |  |
| Strategy 2 | 7,668 | 11.95 | - | - | - | - | - | - |
| Strategy 3 | 7,525 | 11.96 | -143.71 | 0.0104 | -13,840 | 299 | 351 | 455 |
| **Subgroup analysis** |  |  |  |  |  |  |  |  |
| *40-49 years old* |  |  |  |  |  |  |  |  |
| Strategy 2 | 2,202 | 14.12 | - | - | - | - | - | - |
| Strategy 3 | 2,245 | 14.13 | 42.88 | 0.0009 | 47,420 | -29 | -25 | -16 |
| *Men aged 50-74 years old, risk assessment at 35% for Strategy 1* |  |  |  |  |  |  |  |  |
| Strategy 2 | 1,592 | 9.74 | - | - | - | - | - | - |
| Strategy 3 | 1,638 | 9.74 | 46.25 | 0.0012 | 39,851 | -29 | -23 | -11 |
| *Women aged 50-64 years old, risk assessment at 35% for Strategy 1* |  |  |  |  |  |  |  |  |
| Strategy 2 | 3,104 | 10.88 | - | - | - | - | - | - |
| Strategy 3 | 3,093 | 10.88 | -11.23 | 0.0029 | -3,850 | 55 | 70 | 99 |
| *Men aged 50-74 years old, risk assessment at 50% for Strategy 1* |  |  |  |  |  |  |  |  |
| Strategy 2 | 1,592 | 9.74 | - | - | - | - | - | - |
| Strategy 3 | 1,638 | 9.74 | 46.25 | 0.0012 | 39,851 | -29 | -23 | -11 |
| *Women aged 50-64 years old, risk assessment at 50% for Strategy 1* |  |  |  |  |  |  |  |  |
| Strategy 2 | 3,104 | 10.88 | - | - | - | - | - | - |
| Strategy 3 | 3,093 | 10.88 | -11.23 | 0.0029 | -3,850 | 55 | 70 | 99 |
| *Men aged 75-79 years old, risk assessment at 100% for Strategy 1* |  |  |  |  |  |  |  |  |
| Strategy 2 | 799 | 5.07 | - | - | - | - | - | - |
| Strategy 3 | 854 | 5.07 | 54.58 | 0.0006 | 85,795 | -45 | -42 | -35 |
| *Women aged 65-79 years old, risk assessment at 100% for Strategy 1* |  |  |  |  |  |  |  |  |
| Strategy 2 | 1,688 | 6.96 | - | - | - | - | - | - |
| Strategy 3 | 1,703 | 6.96 | 14.83 | 0.0022 | 6,766 | 18 | 29 | 51 |

DXA: dual x-ray absorptiometry, ICER: incremental cost-effectiveness ratio, QALY: quality-adjusted life-year

*99.9% instead of 100% was used as the high value for IDFracture sensitivity due to the incremental QALY between Strategy 2 and Strategy 3 to be less than <0.00001 when sensitivity of IDFracture is 100% and the ICER goes to infinity due to the small incremental QALY.

## Supplementary Table 6. Cost-effectiveness analysis of Strategy 2 vs. Strategy 3 among people with intellectual disabilities with hip fracture

|  | **Mean Cost** | **Mean QALY** | **Incremental Cost** | **Incremental QALY** | **ICER (£/QALY)** | **Incremental net monetary benefit at cost-effectiveness threshold of** | | |
| --- | --- | --- | --- | --- | --- | --- | --- | --- |
|  |  |  |  |  |  | **£15 000/ QALY** | **£20 000/ QALY** | **£30 000/ QALY** |
| **Base case** |  |  |  |  |  |  |  |  |
| Strategy 2 | 1,747 | 12.27 | - | - | - | - | - | - |
| Strategy 3 | 1,785 | 12.27 | 37.92 | 0.0004 | 107,731 | -33 | -31 | -27 |
| **Sensitivity analysis** |  |  |  |  |  |  |  |  |
| *Adherence to osteoporosis treatment -20%* |  |  |  |  |  |  |  |  |
| Strategy 2 | 1,754 | 12.27 | - | - | - | - | - | - |
| Strategy 3 | 1,792 | 12.27 | 37.87 | 0.0003 | 109,245 | -33 | -31 | -27 |
| *Adherence to osteoporosis treatment at 100%* |  |  |  |  |  |  |  |  |
| Strategy 2 | 1,712 | 12.28 | - | - | - | - | - | - |
| Strategy 3 | 1,750 | 12.28 | 37.73 | 0.0008 | 45,782 | -25 | -21 | -13 |
| *Adherence to osteopenia treatment -20%* |  |  |  |  |  |  |  |  |
| Strategy 2 | 1,754 | 12.27 | - | - | - | - | - | - |
| Strategy 3 | 1,791 | 12.27 | 37.59 | 0.0003 | 108,285 | -32 | -31 | -27 |
| *Adherence to osteopenia treatment at 100%* |  |  |  |  |  |  |  |  |
| Strategy 2 | 1,745 | 12.27 | - | - | - | - | - | - |
| Strategy 3 | 1,785 | 12.27 | 39.64 | 0.0004 | 99,846 | -34 | -32 | -28 |
| *Sensitivity of IDFracture for fracture -20%* |  |  |  |  |  |  |  |  |
| Strategy 2 | 1,749 | 12.27 | - | - | - | - | - | - |
| Strategy 3 | 1,785 | 12.27 | 35.76 | 0.0006 | 60,585 | -27 | -24 | -18 |
| *Sensitivity of IDFracture for fracture at 99.9%** |  |  |  |  |  |  |  |  |
| Strategy 2 | 1,744 | 12.27 | - | - | - | - | - | - |
| Strategy 3 | 1,785 | 12.27 | 41.08 | <0.0001 | 26,613,676 | -41 | -41 | -41 |
| *Specificity of IDFracture for fracture -20%* |  |  |  |  |  |  |  |  |
| Strategy 2 | 1,764 | 12.27 | - | - | - | - | - | - |
| Strategy 3 | 1,785 | 12.27 | 21.50 | 0.0004 | 61,080 | -16 | -14 | -11 |
| *Specificity of IDFracture for fracture at 100%* |  |  |  |  |  |  |  |  |
| Strategy 2 | 1,699 | 12.27 | - | - | - | - | - | - |
| Strategy 3 | 1,785 | 12.27 | 86.76 | 0.0004 | 246,494 | -81 | -80 | -76 |
| *Cost of alendrontate -50%* |  |  |  |  |  |  |  |  |
| Strategy 2 | 1,487 | 12.27 | - | - | - | - | - | - |
| Strategy 3 | 1,525 | 12.27 | 37.64 | 0.0004 | 106,941 | -32 | -31 | -27 |
| *Cost of alendrontate +100%* |  |  |  |  |  |  |  |  |
| Strategy 2 | 2,267 | 12.27 | - | - | - | - | - | - |
| Strategy 3 | 2,306 | 12.27 | 38.47 | 0.0004 | 109,311 | -33 | -31 | -28 |
| *Cost of vitamin D and calcium treatment -50%* |  |  |  |  |  |  |  |  |
| Strategy 2 | 1,314 | 12.27 | - | - | - | - | - | - |
| Strategy 3 | 1,351 | 12.27 | 37.16 | 0.0004 | 105,568 | -32 | -30 | -27 |
| *Cost of vitamin D and calcium treatment +100%* |  |  |  |  |  |  |  |  |
| Strategy 2 | 2,615 | 12.27 | - | - | - | - | - | - |
| Strategy 3 | 2,654 | 12.27 | 39.44 | 0.0004 | 112,057 | -34 | -32 | -29 |
| *Cost of DXA -50%* |  |  |  |  |  |  |  |  |
| Strategy 2 | 1,729 | 12.27 | - | - | - | - | - | - |
| Strategy 3 | 1,738 | 12.27 | 8.76 | 0.0004 | 24,899 | -3 | -2 | 2 |
| *Cost of DXA +100%* |  |  |  |  |  |  |  |  |
| Strategy 2 | 1,784 | 12.27 | - | - | - | - | - | - |
| Strategy 3 | 1,881 | 12.27 | 96.23 | 0.0004 | 273,396 | -91 | -89 | -86 |
| *Cost of fracture -50%* |  |  |  |  |  |  |  |  |
| Strategy 2 | 1,715 | 12.27 | - | - | - | - | - | - |
| Strategy 3 | 1,754 | 12.27 | 38.53 | 0.0004 | 109,468 | -33 | -31 | -28 |
| *Cost of fracture +100%* |  |  |  |  |  |  |  |  |
| Strategy 2 | 1,812 | 12.27 | - | - | - | - | - | - |
| Strategy 3 | 1,848 | 12.27 | 36.70 | 0.0004 | 104,258 | -31 | -30 | -26 |
| *Cost of post-fracture -50%* |  |  |  |  |  |  |  |  |
| Strategy 2 | 1,646 | 12.27 | - | - | - | - | - | - |
| Strategy 3 | 1,687 | 12.27 | 40.56 | 0.0004 | 115,250 | -35 | -34 | -30 |
| *Cost of post-fracture +100%* |  |  |  |  |  |  |  |  |
| Strategy 2 | 1,949 | 12.27 | - | - | - | - | - | - |
| Strategy 3 | 1,982 | 12.27 | 32.62 | 0.0004 | 92,693 | -27 | -26 | -22 |
| *10-year time horizon* |  |  |  |  |  |  |  |  |
| Strategy 2 | 914 | 7.18 | - | - | - | - | - | - |
| Strategy 3 | 954 | 7.18 | 40.27 | 0.0001 | 319,747 | -38 | -38 | -36 |
| *Lifetime fracture risk* |  |  |  |  |  |  |  |  |
| Strategy 2 | 3,179 | 12.19 | - | - | - | - | - | - |
| Strategy 3 | 3,183 | 12.19 | 3.17 | 0.0022 | 1,465 | 29 | 40 | 62 |
| **Subgroup analysis** |  |  |  |  |  |  |  |  |
| *40-49 years old* |  |  |  |  |  |  |  |  |
| Strategy 2 | 1,747 | 14.15 | - | - | - | - | - | - |
| Strategy 3 | 1,790 | 14.15 | 43.35 | <0.0001 | 752,122 | -42 | -42 | -42 |
| *Men aged 50-74 years old, risk assessment at 35% for Strategy 1* |  |  |  |  |  |  |  |  |
| Strategy 2 | 1,332 | 9.76 | - | - | - | - | - | - |
| Strategy 3 | 1,373 | 9.76 | 40.83 | 0.0002 | 166,726 | -37 | -36 | -33 |
| *Women aged 50-64 years old, risk assessment at 35% for Strategy 1* |  |  |  |  |  |  |  |  |
| Strategy 2 | 1,651 | 10.94 | - | - | - | - | - | - |
| Strategy 3 | 1,688 | 10.94 | 36.37 | 0.0004 | 102,457 | -31 | -29 | -26 |
| *Men aged 50-74 years old, risk assessment at 50% for Strategy 1* |  |  |  |  |  |  |  |  |
| Strategy 2 | 1,332 | 9.76 | - | - | - | - | - | - |
| Strategy 3 | 1,373 | 9.76 | 40.83 | 0.0002 | 166,726 | -37 | -36 | -33 |
| *Women aged 50-64 years old, risk assessment at 50% for Strategy 1* |  |  |  |  |  |  |  |  |
| Strategy 2 | 1,651 | 10.94 | - | - | - | - | - | - |
| Strategy 3 | 1,688 | 10.94 | 36.37 | 0.0004 | 102,457 | -31 | -29 | -26 |
| *Men aged 75-79 years old, risk assessment at 100% for Strategy 1* |  |  |  |  |  |  |  |  |
| Strategy 2 | 736 | 5.08 | - | - | - | - | - | - |
| Strategy 3 | 779 | 5.08 | 42.37 | 0.0001 | 314,266 | -40 | -40 | -38 |
| *Women aged 65-79 years old, risk assessment at 100% for Strategy 1* |  |  |  |  |  |  |  |  |
| Strategy 2 | 1,054 | 6.99 | - | - | - | - | - | - |
| Strategy 3 | 1,093 | 6.99 | 39.09 | 0.0003 | 148,401 | -35 | -34 | -31 |

DXA: dual x-ray absorptiometry, ICER: incremental cost-effectiveness ratio, QALY: quality-adjusted life-year

*99.9% instead of 100% was used as the high value for IDFracture sensitivity due to the incremental QALY between Strategy 2 and Strategy 3 to be less than <0.00001 when sensitivity of IDFracture is 100% and the ICER goes to infinity due to the small incremental QALY.

# FIGURES

## Supplementary Fig. 1. Decision tree model


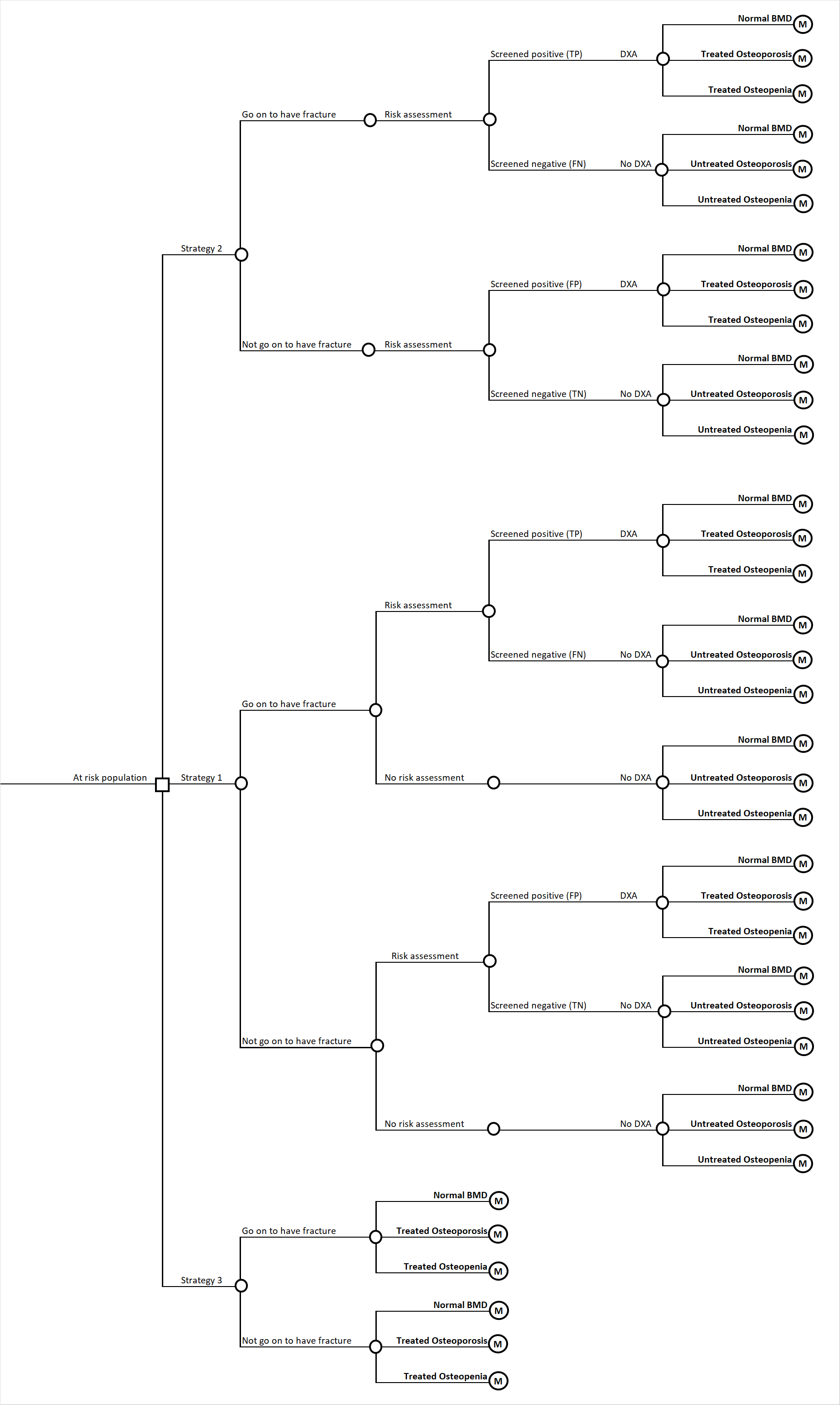


BMD: bone mineral density. DXA: dual-energy X-ray absorptiometry.

## Supplementary Fig. 2. Markov model


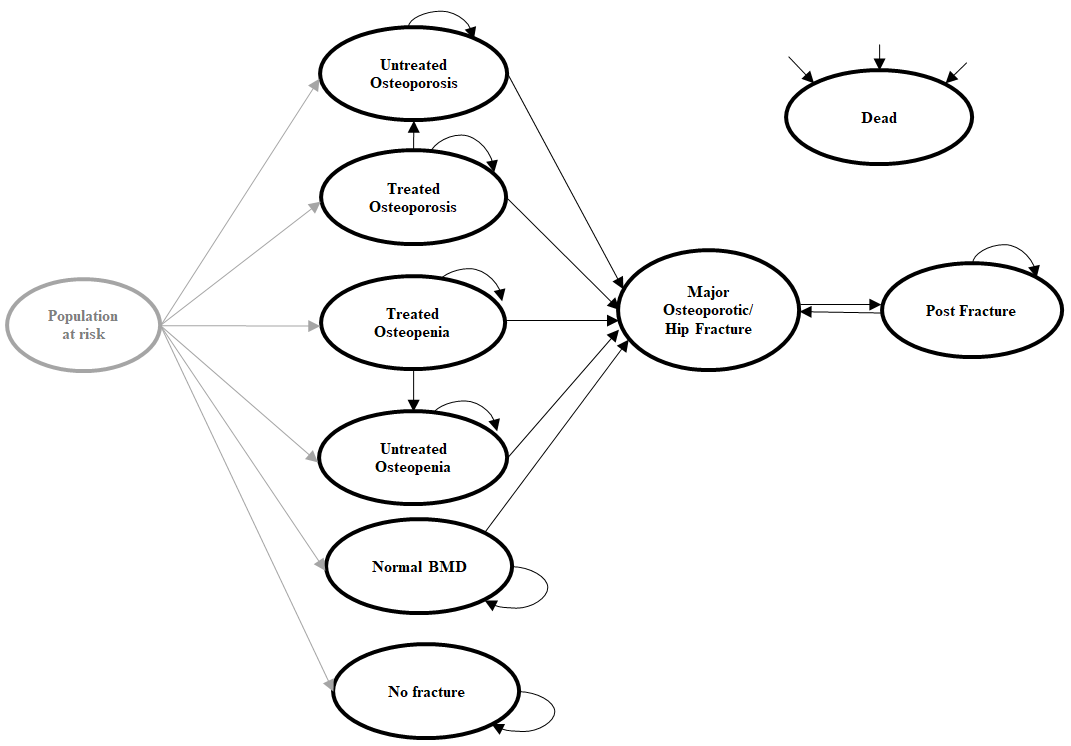


“Population at risk” is not a health state in the Markov model, it represents the movement of people in the decision tree. BMD: bone mineral density.

## Supplementary Fig. 3. Cost-effectiveness analysis of Strategy 2 vs. Strategy 3 among people with intellectual disabilities with major osteoporotic fracture using (a) cost-effectiveness plane and (b) cost-effectiveness acceptability curve.

**
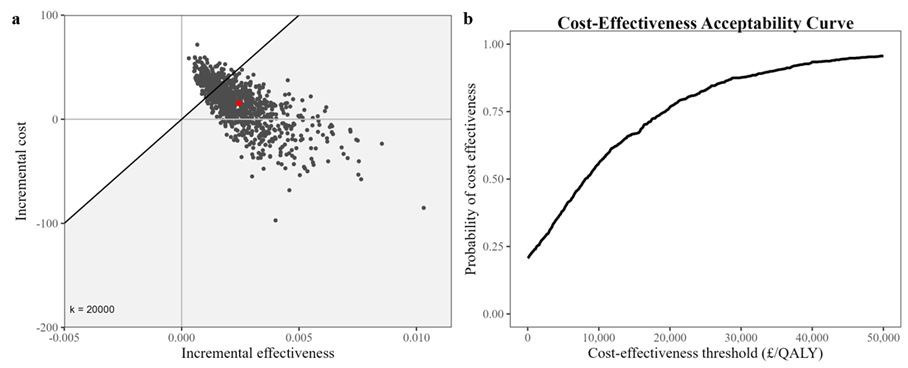
**

## Supplementary Fig. 4. Cost-effectiveness analysis of Strategy 2 vs. Strategy 3 among people with intellectual disabilities with hip fracture using (a) cost-effectiveness plane and (b) cost-effectiveness acceptability curve.


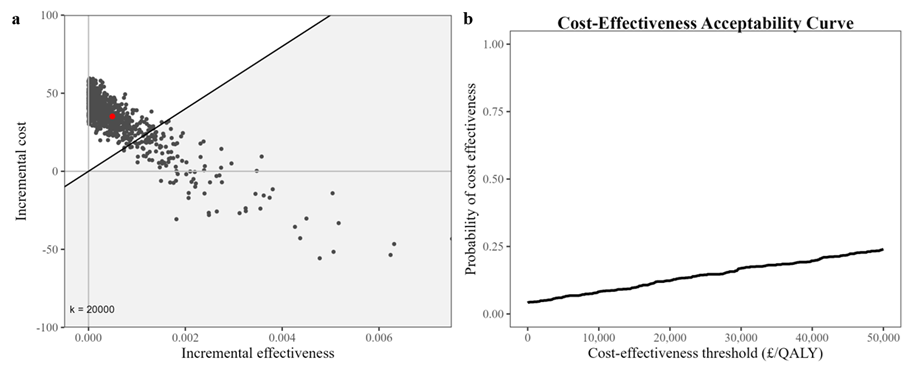


# REFERENCES

1 Frighi V, Smith M, Andrews T, *et al.* Incidence of fractures in people with intellectual disabilities over the life course: a retrospective matched cohort study. *EClinicalMedicine*. 2022;101656. doi: doi.org/10.1016/j.eclinm.2022.101656

2 National Health Service. 2021/22 National Cost Collection Data Publication. 2024. https://www.england.nhs.uk/publication/2021-22-national-cost-collection-data-publication/ (accessed 7 May 2024)

3 NHS Digital. HRG4+ 2018/19 Reference Costs Grouper. 2021. https://digital.nhs.uk/services/national-casemix-office/downloads-groupers-and-tools/costing---hrg4-2018-19-reference-costs-grouper (accessed 23 April 2021)

4 National Health Service. 2019/20 National Cost Collection Data Publication. 2022. https://www.england.nhs.uk/publication/2019-20-national-cost-collection-data-publication/ (accessed 18 March 2022)

5 Jones KC, Weatherly H, Birch S, *et al.* Unit Costs of Health and Social Care 2022. Kent 2023.

6 National Institute for Health and Care Excellence. Chapter 6 GP-led home visits. Emergency and acute medical care in over 16s: service delivery and organisation. NICE guideline 94. 2018. https://www.nice.org.uk/guidance/ng94/evidence/06.gpled-home-visits-pdf-4788818467 (accessed 18 March 2022)

7 Burke É, Carroll R, O’Dwyer M, *et al.* Quantitative examination of the bone health status of older adults with intellectual and developmental disability in Ireland: a cross-sectional nationwide study. *BMJ Open*. 2019;9:e026939.

8 Kanis JA, Johnell O, Oden A, *et al.* FRAX^TM^ and the assessment of fracture probability in men and women from the UK. *Osteoporosis International*. 2008;19:385. doi: 10.1007/S00198-007-0543-5

9 Schuit SCE, Van Der Klift M, Weel AEAM, *et al.* Fracture incidence and association with bone mineral density in elderly men and women: The Rotterdam Study. *Bone*. 2004;34:195–202. doi: 10.1016/j.bone.2003.10.001

10 Jansen J, Bergman G, Huels J, *et al.* The efficacy of bisphosphonates in the prevention of vertebral, hip, and nonvertebral-nonhip fractures in osteoporosis: a network meta-analysis. *Semin Arthritis Rheum*. 2011;40:275–84.

11 Yao P, Bennett D, Mafham M, *et al.* Vitamin D and Calcium for the Prevention of Fracture. *JAMA Netw Open*. 2019;2:e1917789. doi: 10.1001/jamanetworkopen.2019.17789

12 Christensen ER, Clausen A, Petersen TG, *et al.* Excess mortality following a first and subsequent osteoporotic fracture: a Danish nationwide register-based cohort study on the mediating effects of comorbidities. *RMD Open*. 2023;9:e003524. doi: 10.1136/RMDOPEN-2023-003524

13 Kothawala P, Badamgarav E, Ryu S, *et al.* Systematic review and meta-analysis of real-world adherence to drug therapy for osteoporosis. *Mayo Clin Proc*. 2007;82:1493–501.

14 Frighi V, Morovat A, Andrews T, *et al.* Vitamin D, bone mineral density and risk of fracture in people with intellectual disabilities. *Journal of Intellectual Disability Research*. 2019;63:357–67. doi: 10.1111/jir.12581

15 Glover G, Williams R, Heslop P, *et al.* Mortality in people with intellectual disabilities in England. *Journal of Intellectual Disability Research*. 2017;61:62–74. doi: 10.1111/JIR.12314

16 Janssen B, Szende A. Population Norms for the EQ-5D. In: Szende A, Janssen B, Cabases J, eds. *Self-Reported Population Health: An International Perspective based on EQ-5D*. Dordrecht: Springer Netherlands 2014:19–30.

17 Svedbom A, Borgstöm F, Hernlund E, *et al.* Quality of life for up to 18 months after low-energy hip, vertebral, and distal forearm fractures—results from the ICUROS. *Osteoporosis International*. 2018;29:557–66.

18 Jones K, Weatherly H, Birch S, *et al.* Unit costs of health and social care 2022 manual. 2023.

19 NHS Business Services Authority. Prescription Cost Analysis – England – 2021/22. 2022. https://www.nhsbsa.nhs.uk/statistical-collections/prescription-cost-analysis-england/prescription-cost-analysis-england-202122 (accessed 7 May 2024)
