# Supplementary material for: Cost-effectiveness of osteoporotic fracture risk assessment in people with intellectual disabilities: a UK NHS modelling study
Source: BMJ Open. 2026 Apr 17;16(4):e110008. doi: 10.1136/bmjopen-2025-110008 (PMC13110644; doi:10.1136/bmjopen-2025-110008)
Supplement: Supplementary file 2 [file bmjopen-2025-110008.reviewer_comments.pdf]

PEER REVIEW HISTORY

BMJ Open publishes all reviews undertaken for accepted manuscripts. Reviewers are asked to complete a checklist review form and are provided with free text boxes to elaborate on their assessment. These free text comments are reproduced below.

ARTICLE DETAILS

Title (Provisional)

Cost-effectiveness of osteoporotic fracture risk assessment in people with intellectual disabilities: a UK NHS modelling study

Authors

Png, May Ee; Frighi, Valeria; Holt, Tim Adrian; Achana, Felix; Smith, Margaret C; Collins, Gary Stephen; Roast, Jan; Petrou, Stavros

---

VERSION 1 - REVIEW

**Reviewer**	**1**
**Name**	**Watts, Nelson**
**Affiliation**	**Mercy Health Cincinnati, Osteoporosis and Bone Health**
**Date**	**13-Nov-2025**
**COI**	**None**

The authors compared three models for identifying and managing patients with intellectual disabilities. The title says “cost effectiveness of osteoporotic fracture risk assessment…” but they also include management to reduce fracture occurrence. They concluded that “a novel, tailored IDFracture risk score” was less costly and more effective than the other options. As far as I was able to get, I could not see what the “novel, tailored IDFracture risk score” was.

Pages and lines are numbered; thank you. The manuscript is single spaced; I prefer working on double-spaced papers.

The abstract is “bare bones.” The casual reader will not know what is included in “people with intellectual disabilities,” “QFracture,” “current policy thresholds,” “ID Fracture,” or what are the “recommended interventions” in their strategies. The abstract is not very informative and does not draw in the reader.

Page 33, line 10, risk is a gradient, Yes, there is a clear dividing line between the categories of "osteoporosis' and "osteopenia," but this is arbitrary. It is not something to dichotomize. Older patients at the lower end of "osteopenia" are often at higher risk of fracture than young patients at the upper end of "osteoporosis."

Line 15, they justify use of QFracture (which is only available in the UK) instead of FRAX (which is much more widely used and probably better than QFracture) (Kanis et al, J Clin Densitom 2017; 20:444-450) because "it is still named as an option in the 2025 NICE guideline." Just because it is "still named" doesn't mean it would be the best tool for their purposes. If QFracture is removed from the next iteration of the NICE guideline, this work will be wasted.

It turns out the none of the other prediction models for fracture incidence or probability or low BMD work very well in younger postmenopausal women (age 50-65) (see references below)

Page 5, line 30, they tell us that the cohort consisted of people age 40-79 with "ID" but not their sex or age. Almost certainly, any model will work differently in men vs women, young vs old. Without more information about their study population and results of their strategies to identify and treat in subgroups, I can't fathom how to use their results.

Page 5, line 46 and following, treatment is based on a dichotomy that is not defensible. "Calcium and vitamin D" is not "treatment." As a group, patients with BMD in the "osteoporosis" range have a higher risk of fracture than those with "osteopenia;" however, because BMD has a bell-shaped distribution, there are many more individuals with BMD in the "osteopenia range: " more "osteoporotic" fractures occur in patients with BMD in the "osteopenia" range.

Page 5 line, why select alendronate 10 mg daily as "the treatment of choice?" The drug needs to be taken on an empty stomach (usually in the morning) with water only, and a 30 minute wait afterwards before anything other than water – no other medications, juice, coffee, breakfast etc. Many of my patients without "intellectual disabilities" have trouble doing this with the weekly dose of alendronate, much less a daily dose. And what evidence is there for anti-fracture efficacy of any bone-active drug in patients with "intellectual disability?" Almost by definition, patients with "intellectual disability" could not give proper informed consent to be in a placebo-controlled trial.

By the way, clinical trials in patients without “intellectual disability” show a reduction in vertebral fractures of 50-70%, hip fracture 40-50%, but little or no effect of fractures at other sites. Why not use a tool to identify fractures at other sites (and avoid the “noise” of wrist, humerus and pelvic fractures – part of do-called “major osteoporosis-related fractures” that can’t be helped with medication).

Page 6, line 4, they plan for treatments to be given “over a lifetime.” There is general consensus that some (but not all) patients might stop an oral bisphosphonate for a “holiday” of a year or two after 5 years of treatment.

I lost interest at this point.

References

PMID: 37213092

Race and Ethnicity and Fracture Prediction Among Younger Postmenopausal Women in the Women's Health Initiative Study. Crandall CJ, Larson JC, Schousboe JT, et al. .JAMA Intern Med. 2023 Jul 1;183(7):696-704. doi: 10.1001/jamainternmed.2023.1253.PMID: 37213092 Free PMC article.

PMID: 30334182

Predicting Fracture Risk in Younger Postmenopausal Women: Comparison of the Garvan and FRAX Risk Calculators in the Women's Health Initiative Study. Crandall CJ, Larson J, LaCroix A, Cauley JA, et al. J Gen Intern Med. 2019 Feb;34(2):235-242. doi: 10.1007/s11606-018-4696-z. Epub 2018 Oct 17.PMID: 30334182 Free PMC article.

PMID: 25322268

Comparison of fracture risk prediction by the US Preventive Services Task Force strategy and two alternative strategies in women 50-64 years old in the Women's Health Initiative. Crandall CJ, Larson JC, Watts NB, et al. J Clin Endocrinol Metab. 2014 Dec;99(12):4514-22. doi: 10.1210/jc.2014-2332.PMID: 25322268 Free PMC article.

PMID: 24431262

Osteoporosis screening in postmenopausal women 50 to 64 years old: comparison of US Preventive Services Task Force strategy and two traditional strategies in the Women's Health

Initiative. Crandall CJ, Larson J, Gourlay ML, et al. J Bone Miner Res. 2014 Jul;29(7):1661-6. doi: 10.1002/jbmr.2174.PMID: 24431262 Free PMC article.

**Reviewer**	**2**
**Name**	**Roigk, Patrick**
**Affiliation**	**Robert Bosch Hospital**
**Date**	**04-Jan-2026**
**COI**	**None**

Thank you for your work in the field of osteoporosis in people with disability. I dont have any further comments.

**Reviewer**	**3**
**Name**	**Kelson, Zoe**
**Affiliation**	**University of Exeter, Mathematics**
**Date**	**22-Jan-2026**
**COI**	**None**

This study compared the cost-effectiveness of alternative fracture risk assessment strategies for people with intellectual disabilities (ID) aged ≥40 years from a UK National Health Services perspective over a lifetime horizon.

Reviewer comments:

"The results are reported in accordance with the Consolidated Health Economic Evaluation Reporting Standards 2022 (CHEERS 2022) statement[8] for health economic evaluations. "

Thanks for providing a copy of the CHEERS checklist.

Can the modelling methodology applied please be briefly specified in the Abstract?

"In the base-case, S2 (ICER: -£2,568/QALY) was dominant (i.e. less costly and more effective) and S3 (ICER: £1,678/QALY) was cost-effective relative to S1 for major osteoporotic fracture (MOF). For hip fracture, S2 (ICER: £32,116/QALY) and S3 (ICER: £49,536/QALY) were not cost-effective relative to S1 under the NICE-recommended costeffectiveness thresholds."

Can confidence intervals please be reported in the Abstract?

"UK primary care, with data from literature and national databases"

Can the authors please comment on the completeness and coverage of the data used?

"A decision tree with a Markov cohort model was constructed to reflect the options of the three fracture risk assessment strategies, in the primary care setting while modelling

recurring outcomes through time to assess its costs and effects among those aged 40 years old from a UK NHS perspective over a lifetime horizon"

An appropriate modelling methodology has been applied by the authors.

Can equations and model formulation please be provided in the supplementary material?

"Direct healthcare costs and quality-adjusted life-years (QALYs), and incremental cost-effectiveness ratio (ICER)" [Abstract]

and

"Model outcomes were discounted QALYs and direct healthcare costs. The incremental cost-effectiveness ratio (ICER) was calculated as the incremental cost divided by the incremental QALYs and compared between the intervention strategy (i.e. Strategy 2 or Strategy 3) and the comparator strategy (i.e. Strategy 1). A discount rate of 3.5% was applied to outcomes and costs following the NICE guidelines[27]. The NICE cost-effectiveness thresholds of £20,000 per additional QALY[28] and £30,000 per additional QALY[28] were used to determine the costeffectiveness of the fracture risk assessment strategies versus Strategy 1. An additional £15,000 per QALY cost-effectiveness threshold[29] was also used to reflect recent trends in healthcare decision-making. A fracture risk assessment strategy with an ICER below these costeffectiveness thresholds was considered to be cost-effective. The net monetary benefit (NMB) of the fracture risk assessment strategies was computed across different cost-effectiveness thresholds. A positive incremental NMB would indicate that the fracture risk assessment strategy is cost-effective compared with Strategy 1 at the given cost-effectiveness threshold. For completeness, the cost-effectiveness of Strategy 2 versus Strategy 3 was also compared. "

Suitable model metrics have been assessed and compared.

"Deterministic sensitivity analysis and probabilistic sensitivity analyses (PSA) were used to assess the impact of the parameter uncertainties on the cost-effectiveness results."

Rigorous sensitivity analyses have been conducted.

"Subgroup analyses stratified by age group and gender were performed."

Insightful sungroup analyses help to explore the model outcomes.

"Table 1. Base-case cost-effectiveness analysis of the three fracture risk assessment strategies"

and

"Supplementary Table 3. Costs and effects of the fracture risk assessment strategies for major osteoporotic fracture based on sensitivity and subgroup analyses "

and

"Supplementary Table 4. Costs and effects of the fracture risk assessment strategies for hip fracture based on sensitivity and subgroup analyses "

and

"Supplementary Table 5. Cost-effectiveness analysis of Strategy 2 vs. Strategy 3 among people with intellectual disabilities with major osteoporotic fracture"

and

"Supplementary Table 6. Cost-effectiveness analysis of Strategy 2 vs. Strategy 3 among people with intellectual disabilities with hip fracture"

Can the authors please generate and report confidence intervals for all model outputs (e.g. by applying bootstrapping)?

"Supplementary Table 1. Model parameters"

The model inputs and parameter values, with uncertainty/distribution/range and source/citation, have been clearly tabulated.

"Parameters were not always available for people with intellectual disabilities (ID), although ID-specific estimates were used whenever possible. • We used QFracture rather than FRAX as our tool to determine who required a DXA scan for Strategy 1. FRAX has become the more widely used tool, but QFracture is still named as an option in the 2025 NICE guideline. • Other modelling assumptions (healthcare perspective only, lifetime treatment, DXA uptake, linear extrapolation of fracture risk, use of an older QFracture version) may affect generalisability of findings. " [Strengths and Limitations of this Study]

and

"However, this study is not without its limitations. First, many parameters are not based on people with ID but we have tried to use intellectual disability-specific estimates whenever available. One such example is the cost of fractures. [11]Next, we did not include costs from the societal perspective, and this underestimates the additional economic burden of fractures (and the benefits of preventing them) because residential care or supportive living accommodation and individual productivity loss may be cost drivers[34]. We assumed that all people offered DXA scan would receive this investigation, but in fact a proportion of the ID community may not easily tolerate this procedure. Research to investigate more acceptable forms of measurement in this population is a priority. A proportion of people suffering an osteoporotic fracture may have had a previously normal DXA scan, a fact built into our model. We had also assumed treatment over a lifetime due to model constraints, which is not the current practice where the need of continued treatment of bisphosphonate is re-evaluated after five years of bisphosphonate treatment[5]. This would overestimate the cost of osteoporosis treatment. We extrapolated the 10-year fracture risk to a lifetime time horizon linearly in our sensitivity analysis, but the fracture risk is likely to increase exponentially over time[4]. We had used the 2012 version of the QFracture algorithm, so our

computation differed slightly from the latest 2016 version, and we did not have all variables used in the algorithm in our data e.g. family history of osteoporosis, living in a nursing or care home, so our QFracture predicted risks were not identical to those that would have been produced by the risk calculator. Last, we had assumed a constant post-fracture cost even though it is likely to decrease over time"

The authors have provided a Discussion on the study limitations.

VERSION 1 - AUTHOR RESPONSE

REVIEWER 1:

1. The authors compared three models for identifying and managing patients with intellectual disabilities. The title says "cost effectiveness of osteoporotic fracture risk assessment…" but they also include management to reduce fracture occurrence. They concluded that "a novel, tailored IDFracture risk score" was less costly and more effective than the other options. As far as I was able to get, I could not see what the "novel, tailored IDFracture risk score" was.

Response: The evaluation focuses on fracture risk assessment strategies, which necessarily include downstream management following risk identification in order to estimate costs and health outcomes. IDFracture is already described in the Introduction as a fracture risk prediction algorithm developed and externally validated for adults with intellectual disabilities, generating 10-year risks of major osteoporotic and hip fracture (Introduction, paragraph 2). To improve clarity for readers less familiar with the tool, we have revised this section to ensure that the description of IDFracture is clear and prominent. Paragraph 2 now reads: "A new fracture risk prediction tool (IDFracture) for assessing fracture risk in people with ID has been developed and validated (Smith et al, in press)[6]."

2. Pages and lines are numbered; thank you. The manuscript is single spaced; I prefer working on double-spaced papers.

Response: The manuscript and response document have been reformatted to double spacing throughout.

3. The abstract is “bare bones.” The casual reader will not know what is included in “people with intellectual disabilities,” “QFracture,” “current policy thresholds,” “ID Fracture,” or what are the “recommended interventions” in their strategies. The abstract is not very informative and does not draw in the reader.

Response: We have revised the Abstract to improve clarity for readers less familiar with the tools and terminology used. In particular, we have clarified the description of the risk assessment tools and the downstream interventions. The revised text now reads:

> Three strategies were assessed: (S1) Risk assessment using the UK QFracture score; (S2) use of a fracture risk prediction tool specifically developed for adults with intellectual disabilities (IDFracture); and (S3) conducting a one-time dual-energy X-ray absorptiometry (DXA) scan in all. S1 and S2 were followed by DXA scan for those at risk. At-risk individuals received treatment according to UK practice (bisphosphonates plus vitamin D and calcium for osteoporosis, and vitamin D and calcium alone for osteopenia).

4. Page 33, line 10, risk is a gradient, Yes, there is a clear dividing line between the categories of “osteoporosis’ and “osteopenia,” but this is arbitrary. It is not something to dichotomize. Older patients at the lower end of “osteopenia” are often at higher risk of fracture than young patients at the upper end of “osteoporosis.”

Response: We agree that fracture risk is continuous and that the diagnostic categories of osteoporosis and osteopenia are threshold-based classifications rather than biological discontinuities. However, the model was designed to reflect prevailing UK primary care practice and available data within the intellectual disability population, in which treatment

decisions are commonly triggered by DXA T-score thresholds. To improve transparency, we have clarified this in the Methods - Model assumptions section as follows:

> Treatment decisions were modelled according to categorical DXA T-score thresholds (osteoporosis versus osteopenia) to reflect prevailing UK primary care practice and available data within the intellectual disability population, although fracture risk is continuous in reality.

5. Line 15, they justify use of QFracture (which is only available in the UK) instead of FRAX (which is much more widely used and probably better than QFracture) (Kanis et al, J Clin Densitom 2017; 20:444-450) because "it is still named as an option in the 2025 NICE guideline." Just because it is "still named" doesn't mean it would be the best tool for their purposes. If QFracture is removed from the next iteration of the NICE guideline, this work will be wasted.

Response: The choice of QFracture was guided by the objective of modelling fracture risk assessment strategies relevant to UK primary care practice and NICE-recommended pathways at the time the study was designed and conducted. QFracture is embedded within UK primary care systems and is referenced to in NICE fracture risk assessment guidance. The intention of the analysis was not to determine the optimal fracture prediction tool in all settings, but to evaluate implementable strategies within the UK NHS context. Should national guidance evolve in future, the decision-analytic framework developed here could accommodate alternative risk prediction tools without altering the overall model structure. We have clarified this rationale in the Methods - Fracture risk assessment strategies section:

> QFracture was selected to determine eligibility for DXA in Strategy 1 to reflect its use within UK primary care systems and its inclusion in NICE fracture risk assessment

guidance at the time of study design. Although FRAX is also widely used, the objective of this analysis was to evaluate strategies aligned with UK NHS practice.

6. It turns out the none of the other prediction models for fracture incidence or probability or low BMD work very well in younger postmenopausal women (age 50-65) (see references below)

References

PMID: 37213092

Race and Ethnicity and Fracture Prediction Among Younger Postmenopausal Women in the Women's Health Initiative Study. Crandall CJ, Larson JC, Schousboe JT, et al. .JAMA Intern Med. 2023 Jul 1;183(7):696-704. doi: 10.1001/jamainternmed.2023.1253.PMID: 37213092 Free PMC article.

PMID: 30334182

Predicting Fracture Risk in Younger Postmenopausal Women: Comparison of the Garvan and FRAX Risk Calculators in the Women's Health Initiative Study. Crandall CJ, Larson J, LaCroix A, Cauley JA, et al. J Gen Intern Med. 2019 Feb;34(2):235-242. doi: 10.1007/s11606-018-4696-z. Epub 2018 Oct 17.PMID: 30334182 Free PMC article.

PMID: 25322268

Comparison of fracture risk prediction by the US Preventive Services Task Force strategy and two alternative strategies in women 50-64 years old in the Women's Health Initiative. Crandall CJ, Larson JC, Watts NB, et al. J Clin Endocrinol Metab. 2014 Dec;99(12):4514-22. doi: 10.1210/jc.2014-2332.PMID: 25322268 Free PMC article.

PMID: 24431262

Osteoporosis screening in postmenopausal women 50 to 64 years old: comparison of US Preventive Services Task Force strategy and two traditional strategies in the Women's

Health Initiative. Crandall CJ, Larson J, Gourlay ML, et al. J Bone Miner Res. 2014 Jul;29(7):1661-6. doi: 10.1002/jbmr.2174.PMID: 24431262 Free PMC article.

Response: We acknowledge the cited literature indicating that fracture risk prediction tools may have limited discrimination in certain subgroups, including younger postmenopausal women in general population cohorts. However, the referenced studies relate to predictive performance in predominantly US populations without intellectual disabilities. Our study did not aim to assess predictive discrimination, but rather to evaluate the comparative cost-effectiveness of alternative risk assessment strategies within a UK NHS context. IDFracture was developed and externally validated specifically for adults with intellectual disabilities, and subgroup analyses stratified by age and gender were undertaken to explore variation in cost-effectiveness across demographic groups. As the focus of the analysis is economic evaluation rather than external validation of risk prediction tools, no changes to the manuscript were made in response to this comment.

7. Page 5, line 30, they tell us that the cohort consisted of people age 40-79 with "ID" but not their sex or age. Almost certainly, any model will work differently in men vs women, young vs old. Without more information about their study population and results of their strategies to identify and treat in subgroups, I can't fathom how to use their results.

Response: We agree that cost-effectiveness may vary by age and sex and that clearer reporting of cohort characteristics improves interpretability. We have therefore revised the Methods section (Model structure, paragraph 3) to explicitly report the demographic characteristics of the modelled population. The following sentence has been added:

> The modelled cohort had a mean age of 51.9 years and comprised 50% men and 50% women.

In addition, we have clarified the subgroup analyses section to emphasise that age- and sex-stratified analyses were undertaken. The first sentence of the Subgroup analyses section has been revised to:

> Subgroup analyses stratified by age group and gender were performed to explore heterogeneity in cost-effectiveness across demographic groups.

8. Page 5, line 46 and following, treatment is based on a dichotomy that is not defensible. “Calcium and vitamin D” is not “treatment.” As a group, patients with BMD in the “osteoporosis” range have a higher risk of fracture than those with “osteopenia;” however, because BMD has a bell-shaped distribution, there are many more individuals with BMD in the “osteopenia range: ” more “osteoporotic” fractures occur in patients with BMD in the “osteopenia” range.

Response: We agree that fracture risk is continuous and that many fractures occur among individuals with BMD values in the osteopenic range due to the underlying population distribution. The model does not assume a biological discontinuity between osteoporosis and osteopenia but rather reflects prevailing UK primary care practice and guideline-based management pathways, in which DXA T-score thresholds commonly inform treatment decisions. In this context, “treatment” refers to management according to common UK practice, particularly at the time we designed the study, where individuals with osteoporosis typically receive bisphosphonates alongside vitamin D and calcium, while those with osteopenia may receive vitamin D and calcium supplementation alone. This assumption was also necessary as we do not have sufficient data on the BMD distribution of the ID population to model re-estimation of fracture risk using BMD values from DXA scans (which is ideal practice). This pragmatic approach is therefore defensible, and is conservative, in that it is likely to under- rather than over-estimate the benefit of screening the ID population for

fracture risk, if only those with osteoporosis receive bisphosphonates. We have revised the Methods (Model structure, paragraph 4) as follows:

> Those with osteoporosis were treated using bisphosphonates in addition to vitamin D and calcium while those with osteopenia were treated with vitamin D and calcium alone in line with common UK practice.

In addition, the Model assumptions section now explicitly states that treatment decisions were modelled according to categorical DXA T-score thresholds to reflect prevailing UK primary care practice and available data within the intellectual disability population, rather than assuming fracture risk to be dichotomous (please refer to comment #4).

9. Page 5 line, why select alendronate 10 mg daily as "the treatment of choice?" The drug needs to be taken on an empty stomach (usually in the morning) with water only, and a 30 minute wait afterwards before anything other than water – no other medications, juice, coffee, breakfast etc. Many of my patients without "intellectual disabilities" have trouble doing this with the weekly dose of alendronate, much less a daily dose. And what evidence is there for anti-fracture efficacy of any bone-active drug in patients with "intellectual disability?" Almost by definition, patients with "intellectual disability" could not give proper informed consent to be in a placebo-controlled trial.

Response: Alendronate was selected in line with NICE guidance, which recommends modelling cost-effectiveness using the lowest available cost bisphosphonate as representative of first-line pharmacological treatment for osteoporosis in UK practice. The intention was not to imply that daily dosing is universally optimal for individuals with intellectual disabilities, but to reflect the standard first-line oral bisphosphonate pathway used in UK primary care economic evaluations.

We recognise that adherence to oral bisphosphonates can be challenging in routine practice, including among individuals without intellectual disabilities. This uncertainty was explored in deterministic sensitivity analyses varying adherence parameters.

With respect to anti-fracture efficacy, randomised controlled trials specifically in people with intellectual disabilities are not available. As is standard in economic modelling, treatment efficacy estimates were derived from general population trials, assuming that the biological effect of bisphosphonates on bone remodelling does not depend on cognitive status. As acknowledged in the Limitations section of the Discussion, several model parameters were not ID-specific, although intellectual disability-specific estimates were used whenever available.

10. By the way, clinical trials in patients without "intellectual disability" show a reduction in vertebral fractures of 50-70%, hip fracture 40-50%, but little or no effect of fractures at other sites. Why not use a tool to identify fractures at other sites (and avoid the "noise" of wrist, humerus and pelvic fractures – part of do-called "major osteoporosis-related fractures" that can't be helped with medication).

Response: We acknowledge that anti-fracture efficacy of bisphosphonates varies by fracture site, with the strongest evidence for vertebral and hip fracture reduction. However, major osteoporotic fracture (MOF), comprising vertebral, hip, wrist and humerus fractures, is a standard composite outcome used in fracture risk prediction tools (including QFracture and IDFracture) and in guideline-recommended treatment thresholds. The use of MOF therefore allows alignment with established risk assessment frameworks and policy pathways. Importantly, hip fracture was also modelled and analysed separately, and results for hip fracture are reported independently in both the main analysis and subgroup analyses. This enables interpretation of cost-effectiveness for the clinically most serious fracture type while maintaining comparability with established composite risk thresholds.

11. Page 6, line 4, they plan for treatments to be given “over a lifetime.” There is general consensus that some (but not all) patients might stop an oral bisphosphonate for a “holiday” of a year or two after 5 years of treatment.

Response: We agree that in current clinical practice, continuation of oral bisphosphonate therapy is typically re-evaluated after approximately five years and that some patients may have a treatment “holiday.” In the model, treatment was assumed to continue over a lifetime for structural simplicity and due to limitations in modelling time-varying treatment discontinuation patterns. This assumption is explicitly acknowledged in the Limitations section of the Discussion, where we state that lifetime treatment is not reflective of current practice and may overestimate the cost of osteoporosis treatment. We have retained this transparent limitation rather than introducing additional structural assumptions that could not be robustly parameterised in the intellectual disability population.

REVIEWER 2:

1. Thank you for your work in the field of osteoporosis in people with disability. I don’t have any further comments.

Response: We thank the reviewer for their positive feedback and have no further response to this comment.

REVIEWER 3:

1. Can the modelling methodology applied please be briefly specified in the Abstract?

Response: The modelling methodology is already specified in the Abstract. In particular, the Design field states: “Cost-effectiveness analysis using a lifetime decision-analytical model,” and the Setting field specifies the UK primary care context and data sources.

2. "In the base-case, S2 (ICER: -£2,568/QALY) was dominant (i.e. less costly and more effective) and S3 (ICER: £1,678/QALY) was cost-effective relative to S1 for major

osteoporotic fracture (MOF). For hip fracture, S2 (ICER: £32,116/QALY) and S3 (ICER: £49,536/QALY) were not cost-effective relative to S1 under the NICE-recommended costeffectiveness thresholds."

Can confidence intervals please be reported in the Abstract?

Response: Confidence intervals are not routinely reported for incremental cost-effectiveness ratios (ICERs) because ICERs are ratio measures with non-standard and potentially undefined distributions. Uncertainty in the cost-effectiveness results was instead assessed using probabilistic sensitivity analysis and is summarised through cost-effectiveness planes and acceptability curves in the Results section. We have therefore not added confidence intervals for ICERs to the Abstract.

3. "UK primary care, with data from literature and national databases"

Can the authors please comment on the completeness and coverage of the data used?

Response: Data completeness and coverage are described in the Methods section of the main text. In particular, we state that the Hospital Episode Statistics dataset contains hospital records for the majority of patients attending NHS secondary care in England, and that CPRD Aurum captures data from over 40 million patients, including approximately 13 million current patients (around 20% of the UK population), across over 1,000 general practices using EMIS Web® software.

4. Can equations and model formulation please be provided in the supplementary material?

Response: The analysis used a standard decision tree followed by a state-transition (Markov cohort) model, implemented using transition probabilities, costs, and utilities rather than novel mathematical equations. The model structure is presented schematically in the Supplementary Figures, and all parameter values and assumptions are detailed in the Supplementary Tables. As no regression-based or analytical equations were derived as part of the modelling, there are no additional equations to report.

5. "Table 1. Base-case cost-effectiveness analysis of the three fracture risk assessment strategies"

   and

   "Supplementary Table 3. Costs and effects of the fracture risk assessment strategies for major osteoporotic fracture based on sensitivity and subgroup analyses "

   and

   "Supplementary Table 4. Costs and effects of the fracture risk assessment strategies for hip fracture based on sensitivity and subgroup analyses "

   and

   "Supplementary Table 5. Cost-effectiveness analysis of Strategy 2 vs. Strategy 3 among people with intellectual disabilities with major osteoporotic fracture"

   and

   "Supplementary Table 6. Cost-effectiveness analysis of Strategy 2 vs. Strategy 3 among people with intellectual disabilities with hip fracture"

   Can the authors please generate and report confidence intervals for all model outputs (e.g. by applying bootstrapping)?

Response: Conventional confidence intervals are not routinely reported for incremental cost-effectiveness ratios in model-based economic evaluations because results represent expected values from a decision model rather than estimates derived from repeated data sampling. Good practice in economic modelling emphasises the examination and reporting of uncertainty using probabilistic sensitivity analysis (PSA), which characterises parameter uncertainty and supports decision uncertainty interpretation. PSA results are appropriately summarised using cost-effectiveness planes and cost-effectiveness acceptability curves, as recommended by methodological guidelines such as the ISPOR-SMDM Modeling Good Research Practices Task Force for characterising uncertainty in decision-analytic models.[1]

1. Briggs AH, Weinstein MC, Fenwick EAL, Karnon J, Sculpher MJ, Paltiel AD. Model Parameter Estimation and Uncertainty Analysis: A Report of the ISPOR-SMDM Modeling Good Research Practices Task Force Working Group–6: A Report of the ISPOR-SMDM Modeling Good Research Practices Task Force Working Group–6. Medical Decision Making. 2012;32(5):722-732. doi:10.1177/0272989X12458348.

VERSION 2 - REVIEW

**Reviewer**	**3**
**Name**	**Kelson, Zoe**
**Affiliation**	**University of Exeter, Mathematics**
**Date**	**19-Mar-2026**
**COI**	

Thanks to the authors for their responses. The following reviewer requests are raised:

Author response:

"The analysis used a standard decision tree followed by a state-transition (Markov cohort) model, implemented using transition probabilities, costs, and utilities rather than novel mathematical equations. The model structure is presented schematically in the Supplementary Figures, and all parameter values and assumptions are detailed in the Supplementary Tables. As no regression-based or analytical equations were derived as part of the modelling, there are no additional equations to report."

Reviewer request:

Can the formulation of the state-transition model please be specified in the supplementary material (i.e. with equations provided)? This will support transparency and reproducibility of the methodology applied.

Author response:

"Conventional confidence intervals are not routinely reported for incremental costeffectiveness ratios in model-based economic evaluations because results represent expected values from a decision model rather than estimates derived from repeated data sampling. Good practice in economic modelling emphasises the examination and reporting of uncertainty using probabilistic sensitivity analysis (PSA), which characterises parameter uncertainty and supports decision uncertainty interpretation. PSA results are appropriately summarised using cost-effectiveness planes and cost-effectiveness acceptability curves, as

recommended by methodological guidelines such as the ISPOR-SMDM Modeling Good Research Practices Task Force for characterising uncertainty in decision-analytic models"

Reviewer request:

ICERs are point estimates, and should ideally be reported with confidence intervals to accurately communicate uncertainty in the model outputs. In addition to the rigorous PSA undertaken by the authors, can they please also revisit this comment regarding the generation of CIs (by applying bootstrapping, for example)?
